# Supplementary figures and images for: ERK5 suppression overcomes FAK inhibitor resistance in mutant KRAS-driven non-small cell lung cancer
Source: EMBO Mol Med. 2024 Sep 13;16(10):2402–26. doi: 10.1038/s44321-024-00138-7 (PMC11473843; doi:10.1038/s44321-024-00138-7)

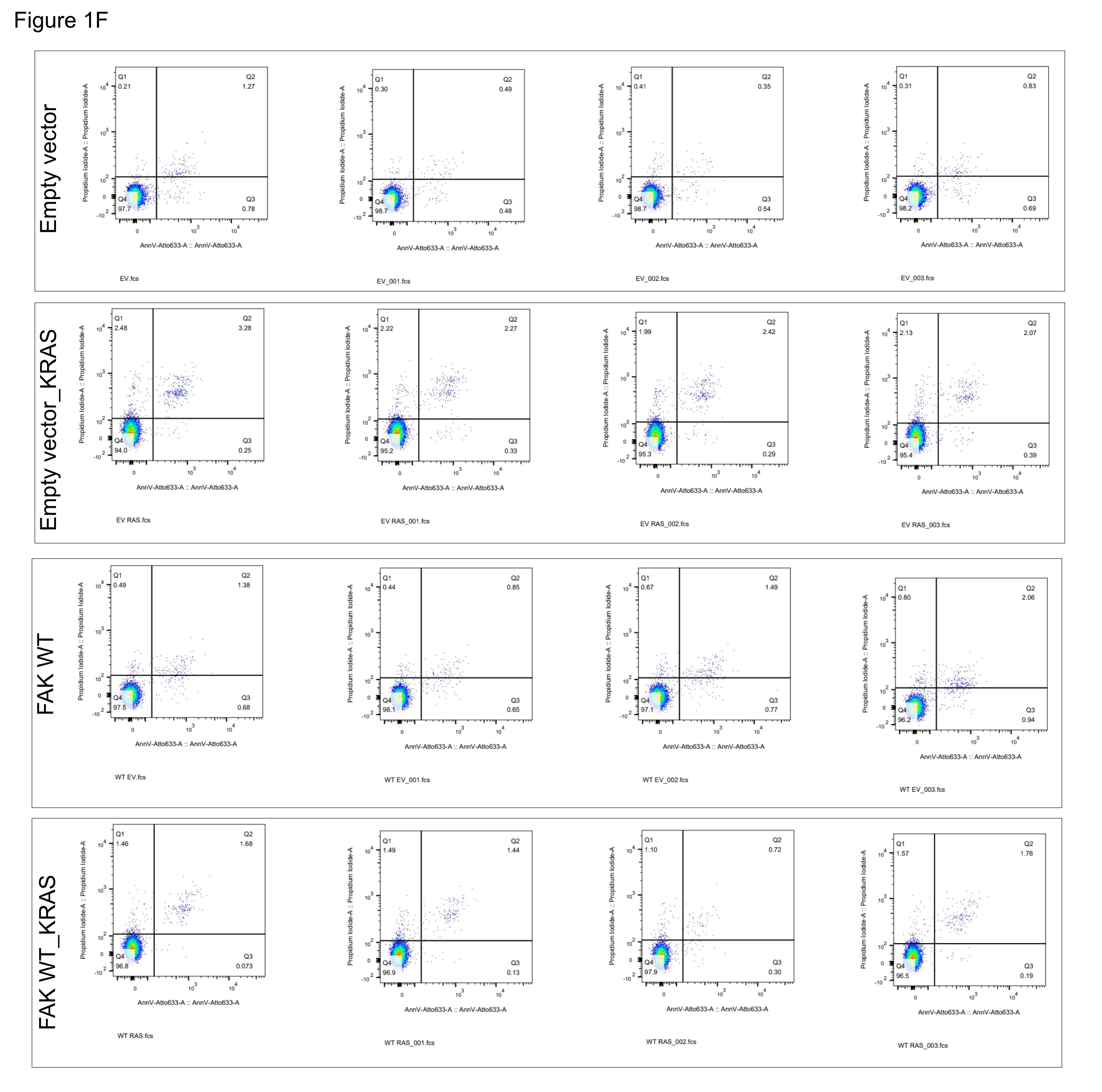

Supplement: Supplementary file 2 — Source data Fig. 1 [file 44321_2024_138_MOESM2_ESM.zip › Figure 1/1F/Fig. 1F- Cell death fcs panels.jpg]

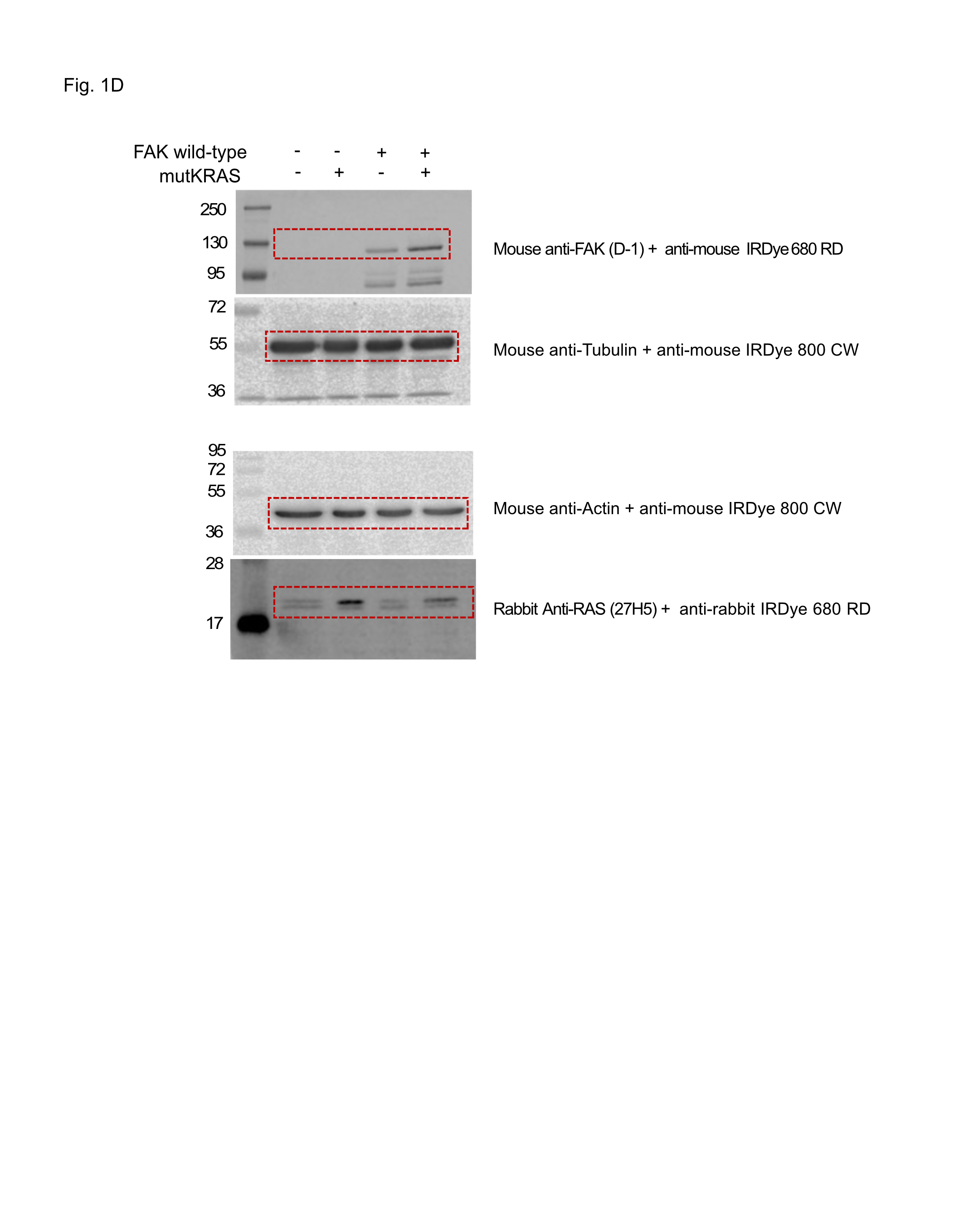

Supplement: Supplementary file 2 — Source data Fig. 1 [file 44321_2024_138_MOESM2_ESM.zip › Figure 1/1D/Fig. 1D-immunoblot.tiff]

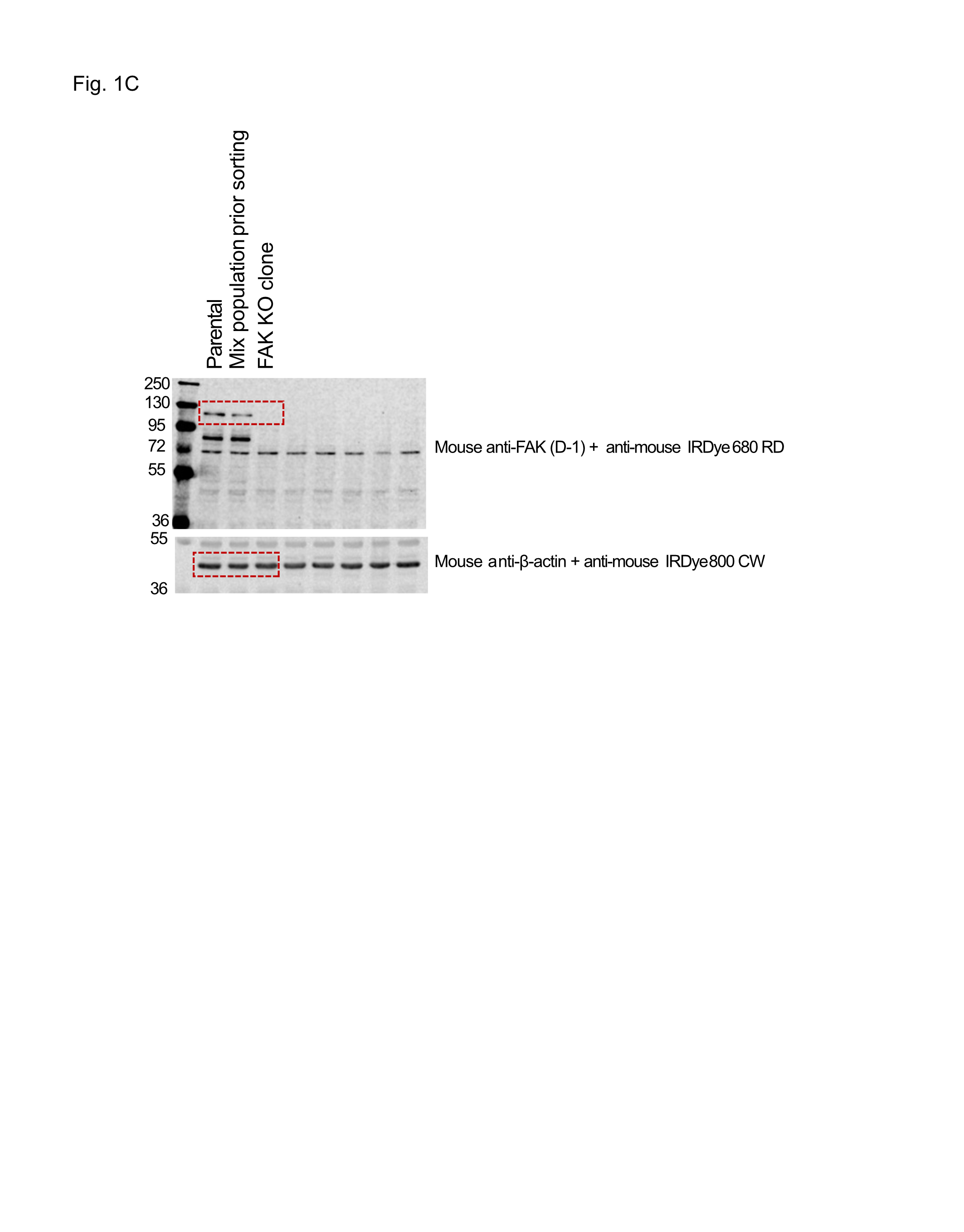

Supplement: Supplementary file 2 — Source data Fig. 1 [file 44321_2024_138_MOESM2_ESM.zip › Figure 1/1C/Fig. 1C-immunoblot.tiff]

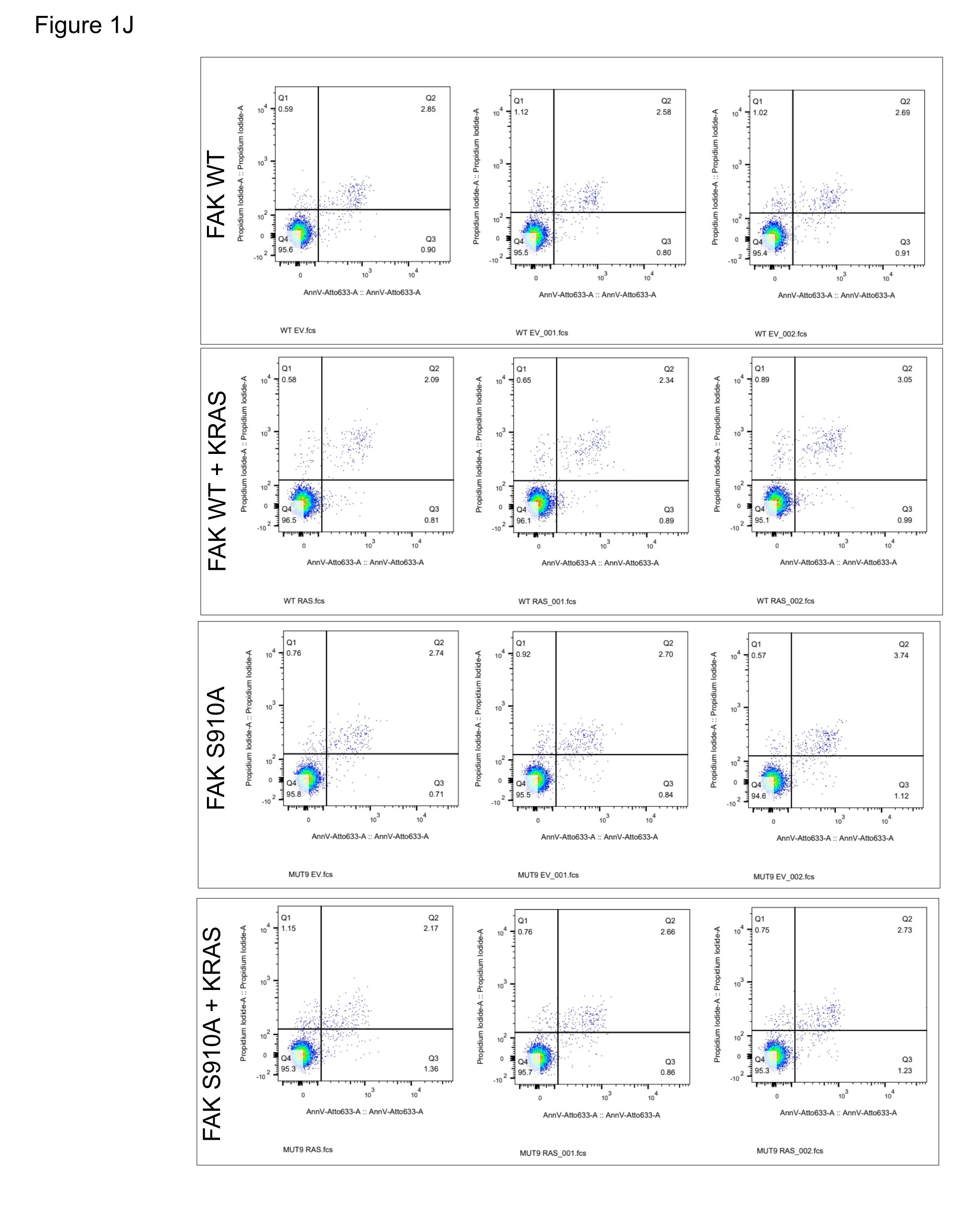

Supplement: Supplementary file 2 — Source data Fig. 1 [file 44321_2024_138_MOESM2_ESM.zip › Figure 1/1J/Fig. 1J_S910A-fcs panels.jpg]

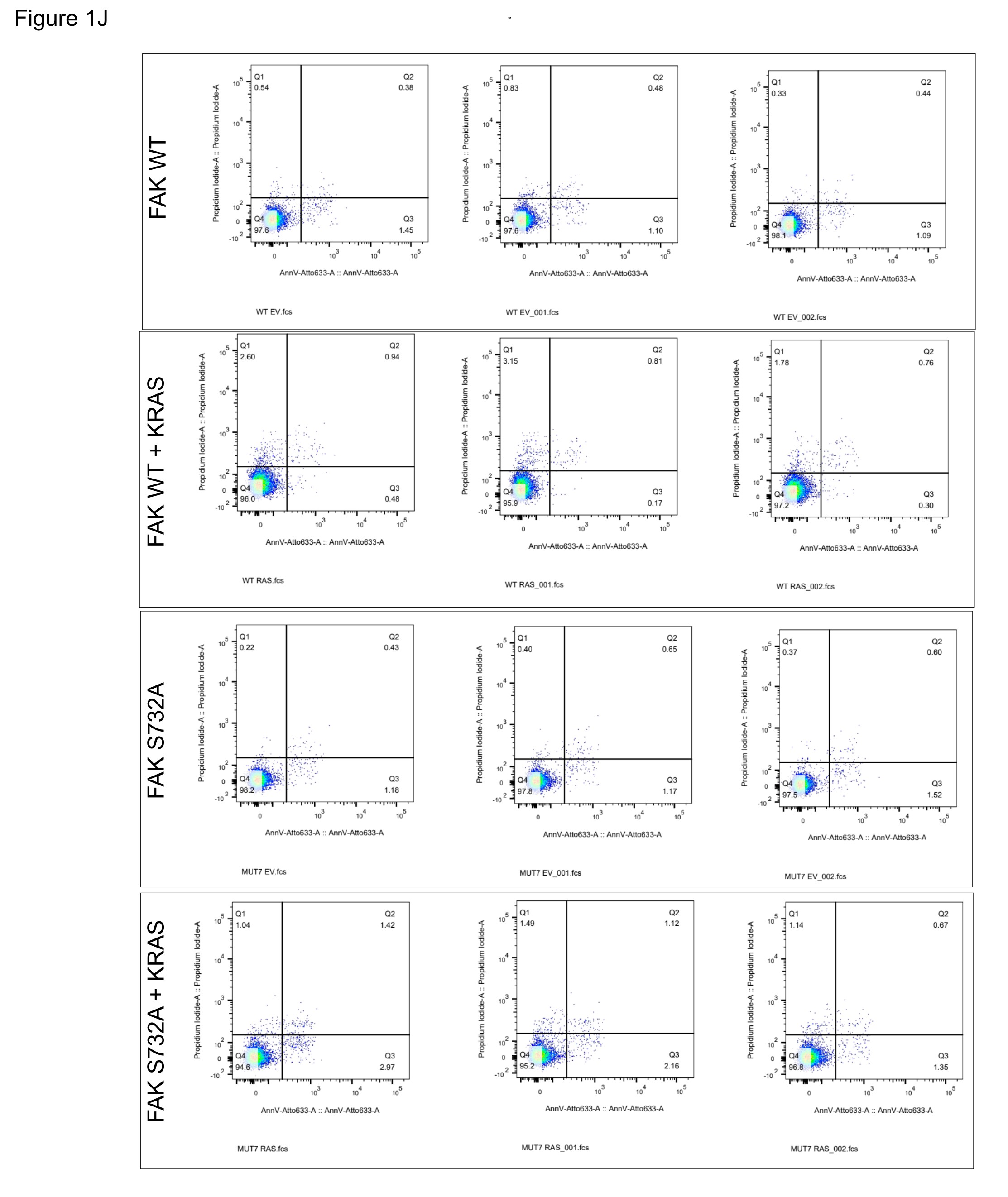

Supplement: Supplementary file 2 — Source data Fig. 1 [file 44321_2024_138_MOESM2_ESM.zip › Figure 1/1J/Fig. 1J_S732A-fcs panels.jpg]

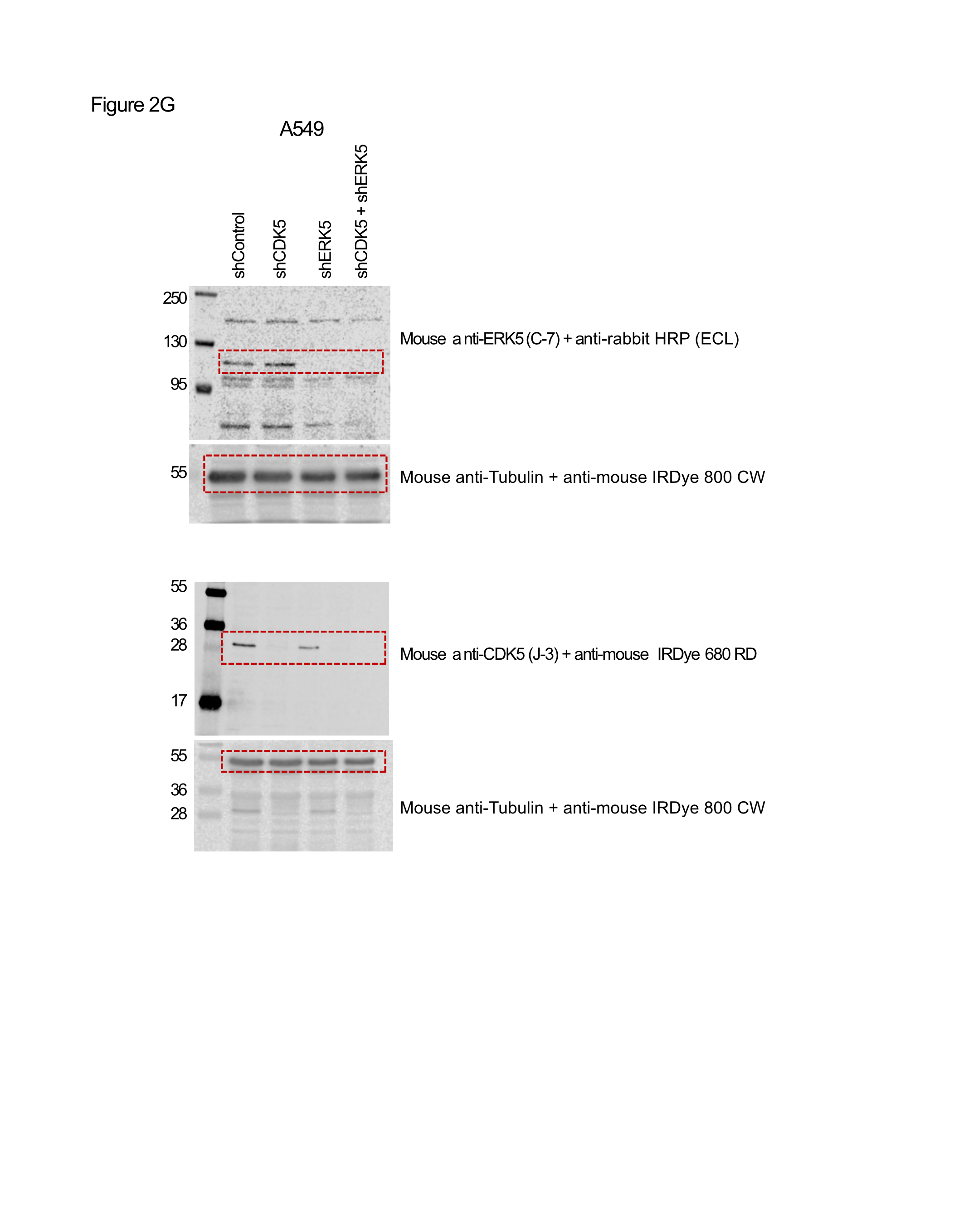

Supplement: Supplementary file 3 — Source data Fig. 2 [file 44321_2024_138_MOESM3_ESM.zip › Figure 2/2G/Fig. 2G-immunoblot.tiff]

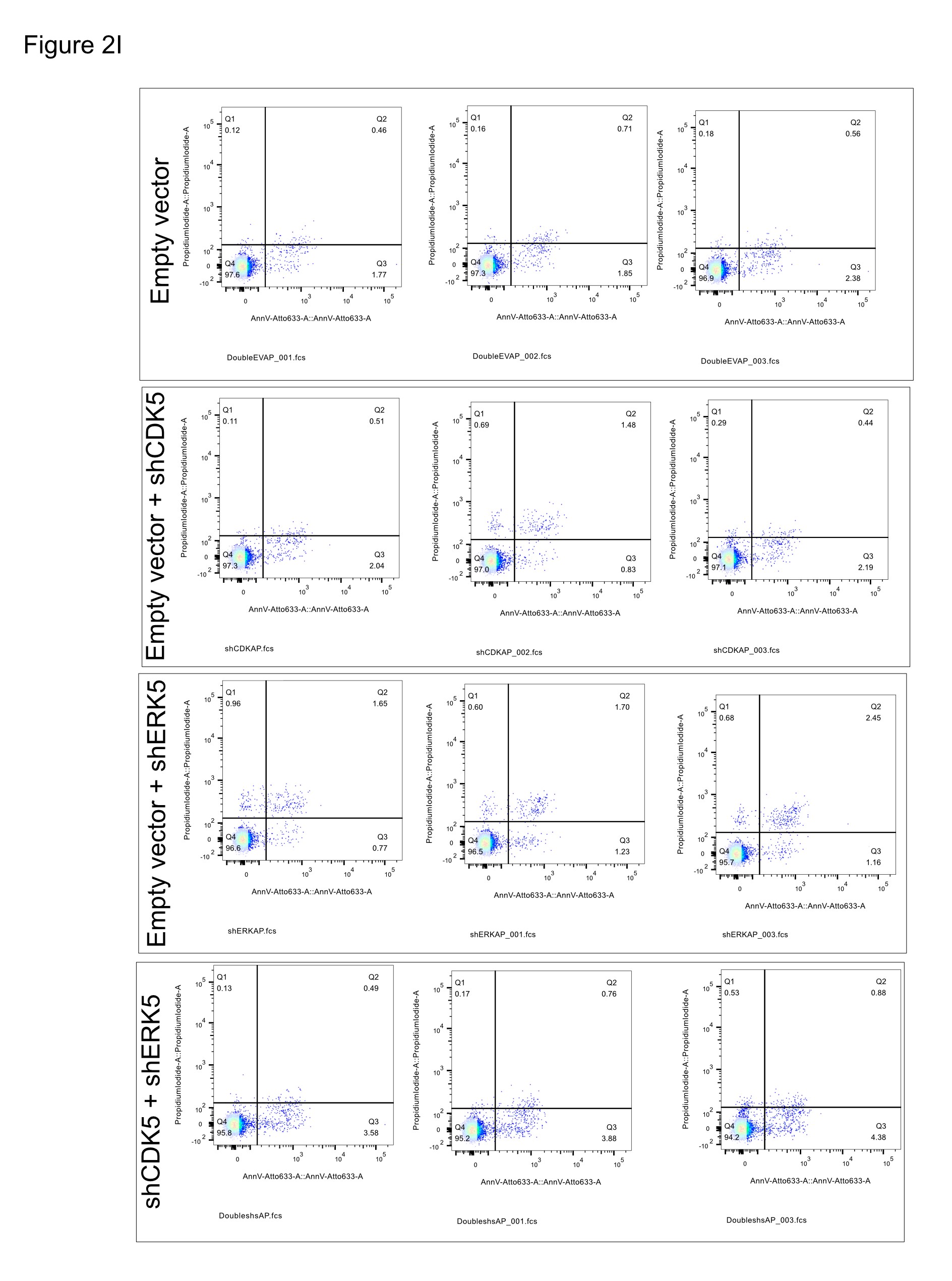

Supplement: Supplementary file 3 — Source data Fig. 2 [file 44321_2024_138_MOESM3_ESM.zip › Figure 2/2I/Fig. 2I-fcs panels.jpg]

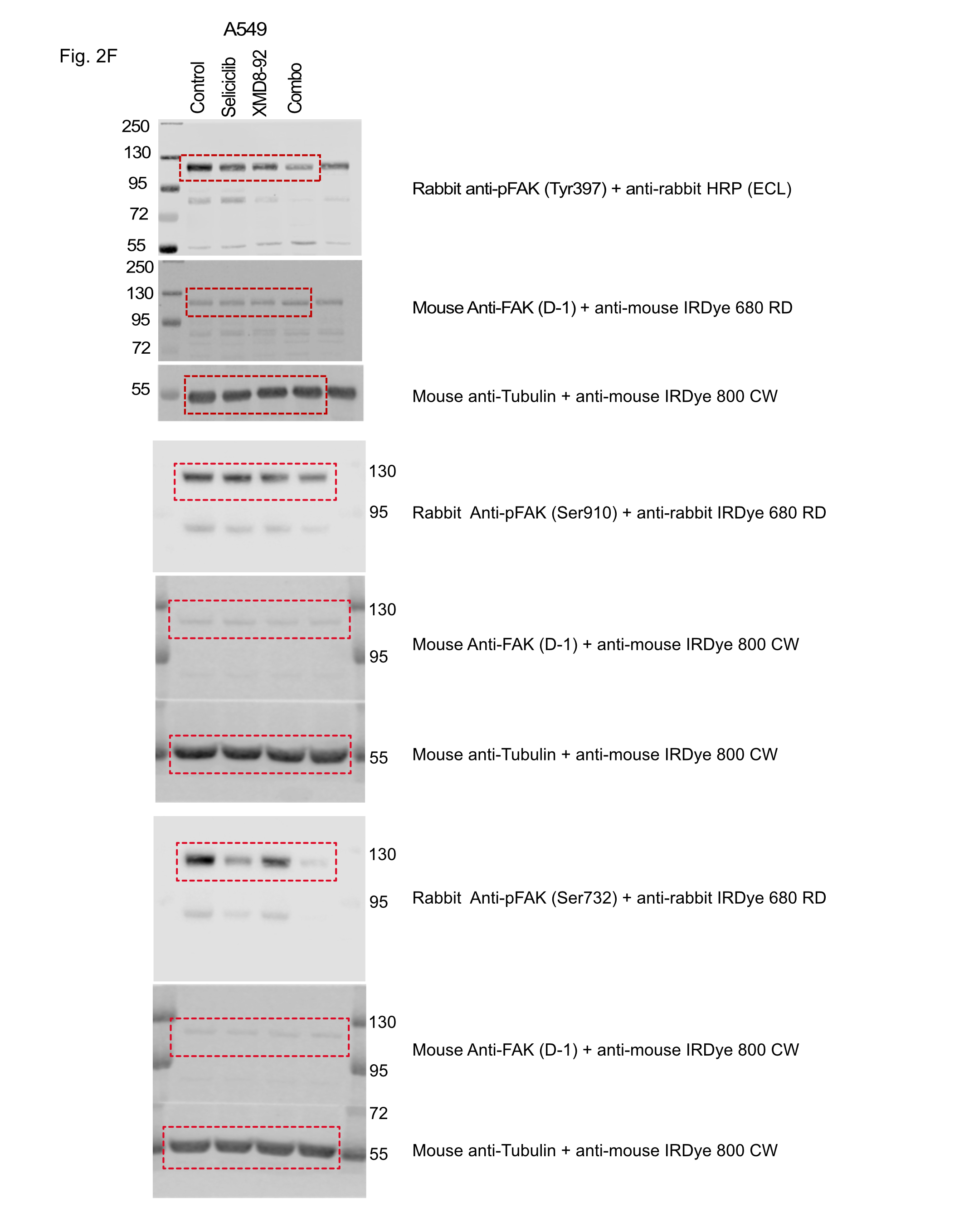

Supplement: Supplementary file 3 — Source data Fig. 2 [file 44321_2024_138_MOESM3_ESM.zip › Figure 2/2F/Fig. 2F.tiff]

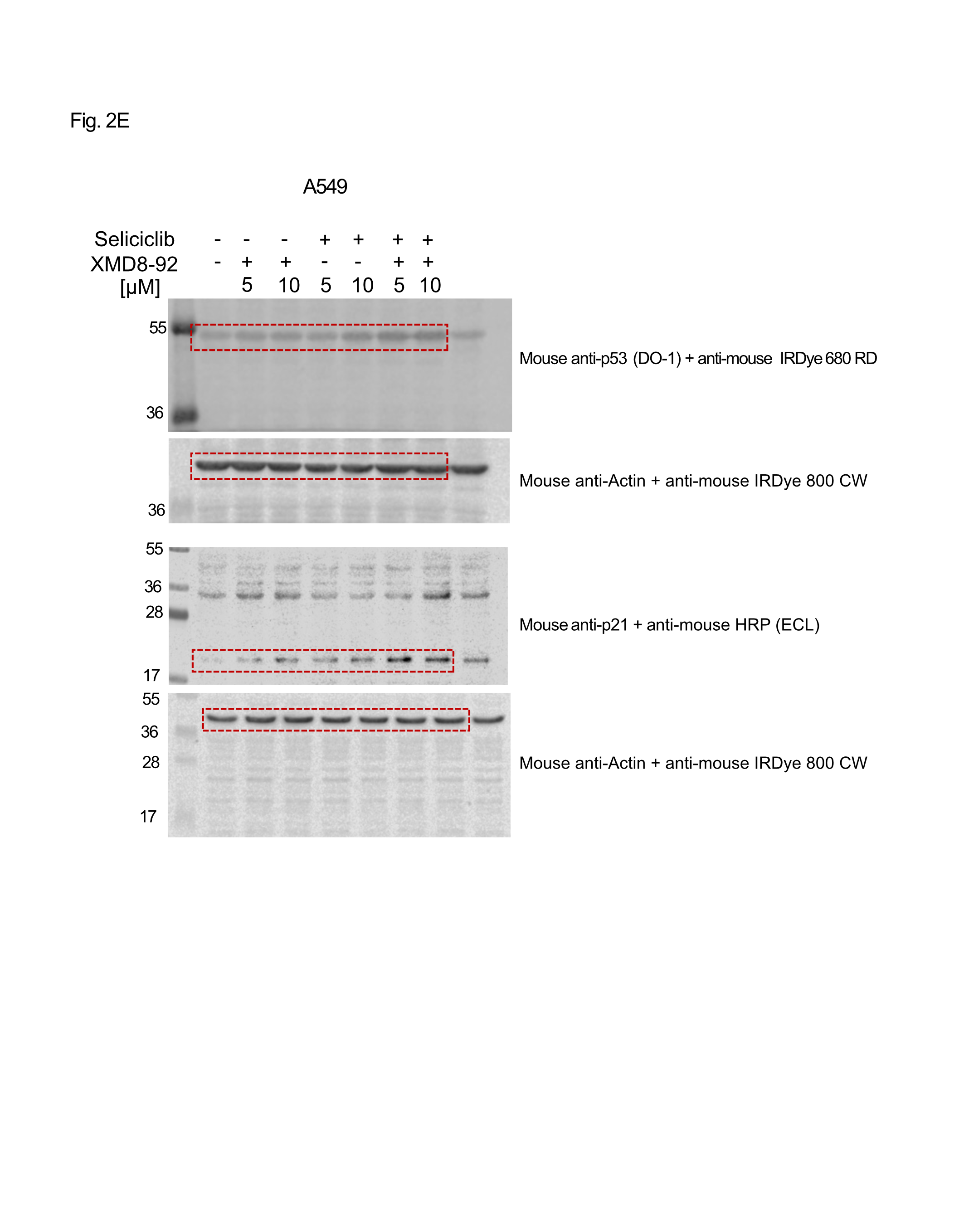

Supplement: Supplementary file 3 — Source data Fig. 2 [file 44321_2024_138_MOESM3_ESM.zip › Figure 2/2E/Fig. 2E-immunoblot.tiff]

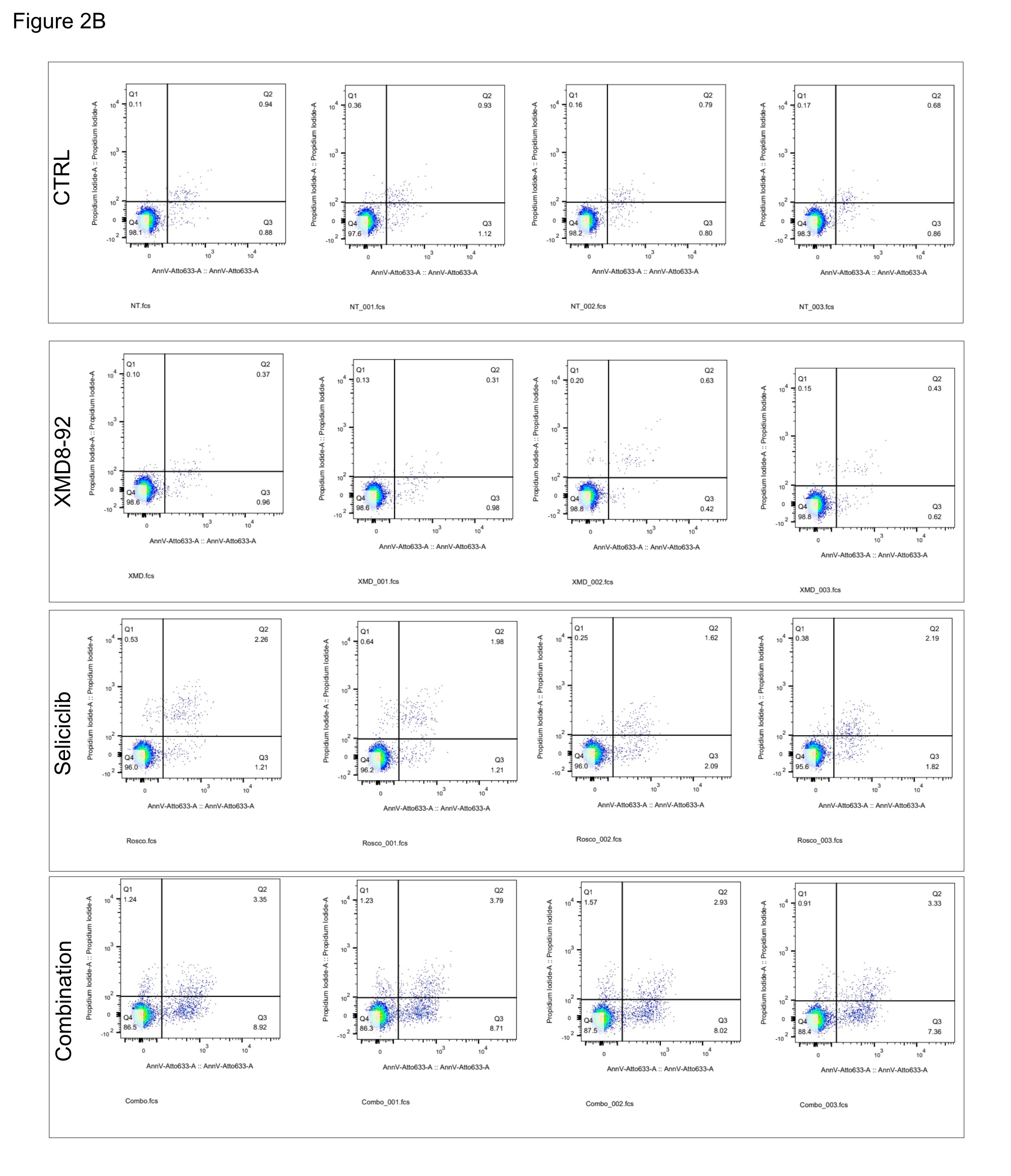

Supplement: Supplementary file 3 — Source data Fig. 2 [file 44321_2024_138_MOESM3_ESM.zip › Figure 2/2B/Fig. 2B-fcs panels.jpg]

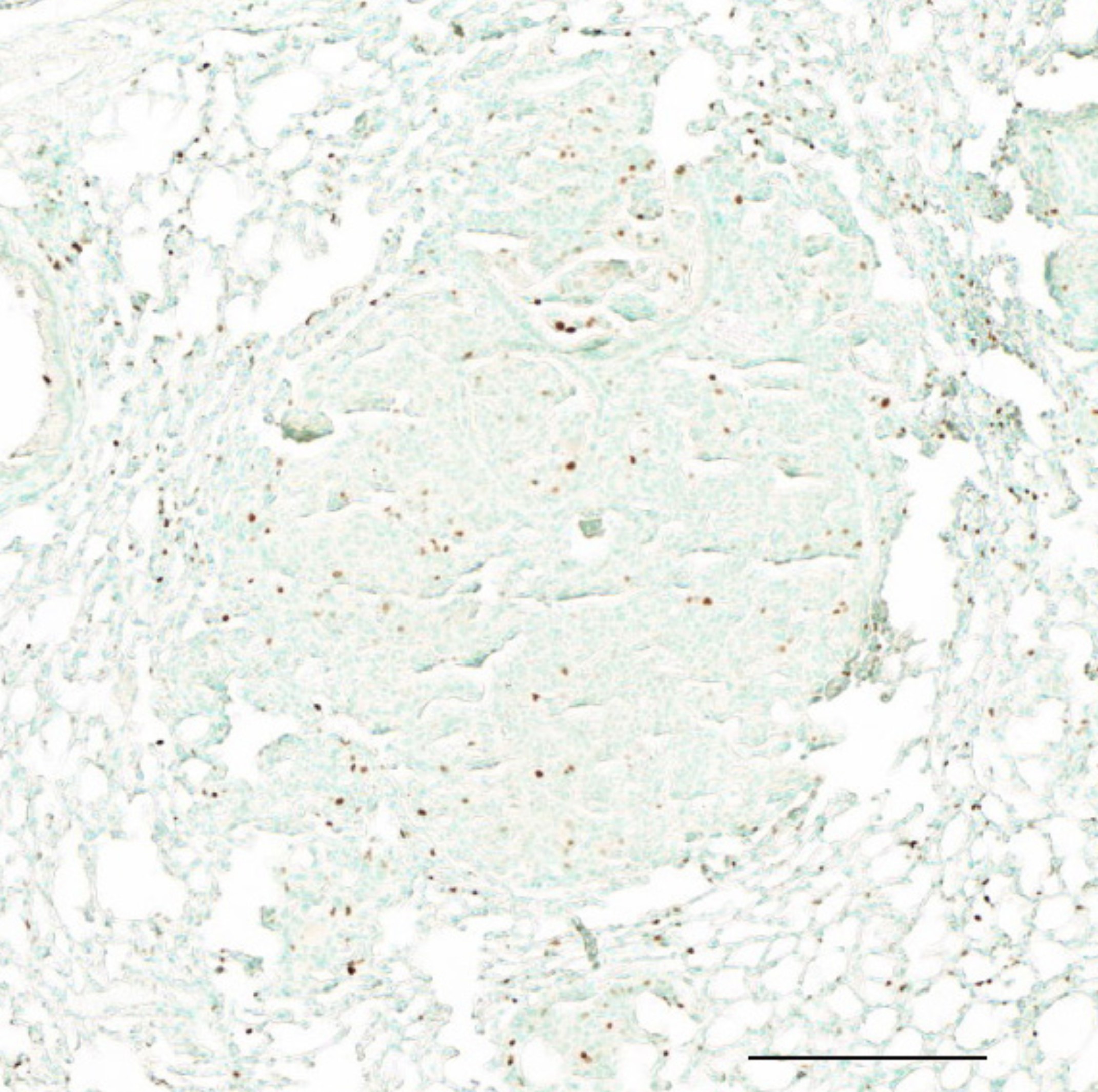

Supplement: Supplementary file 4 — Source data Fig. 3 [file 44321_2024_138_MOESM4_ESM.zip › Figure 3/3C/Combination.jpg]

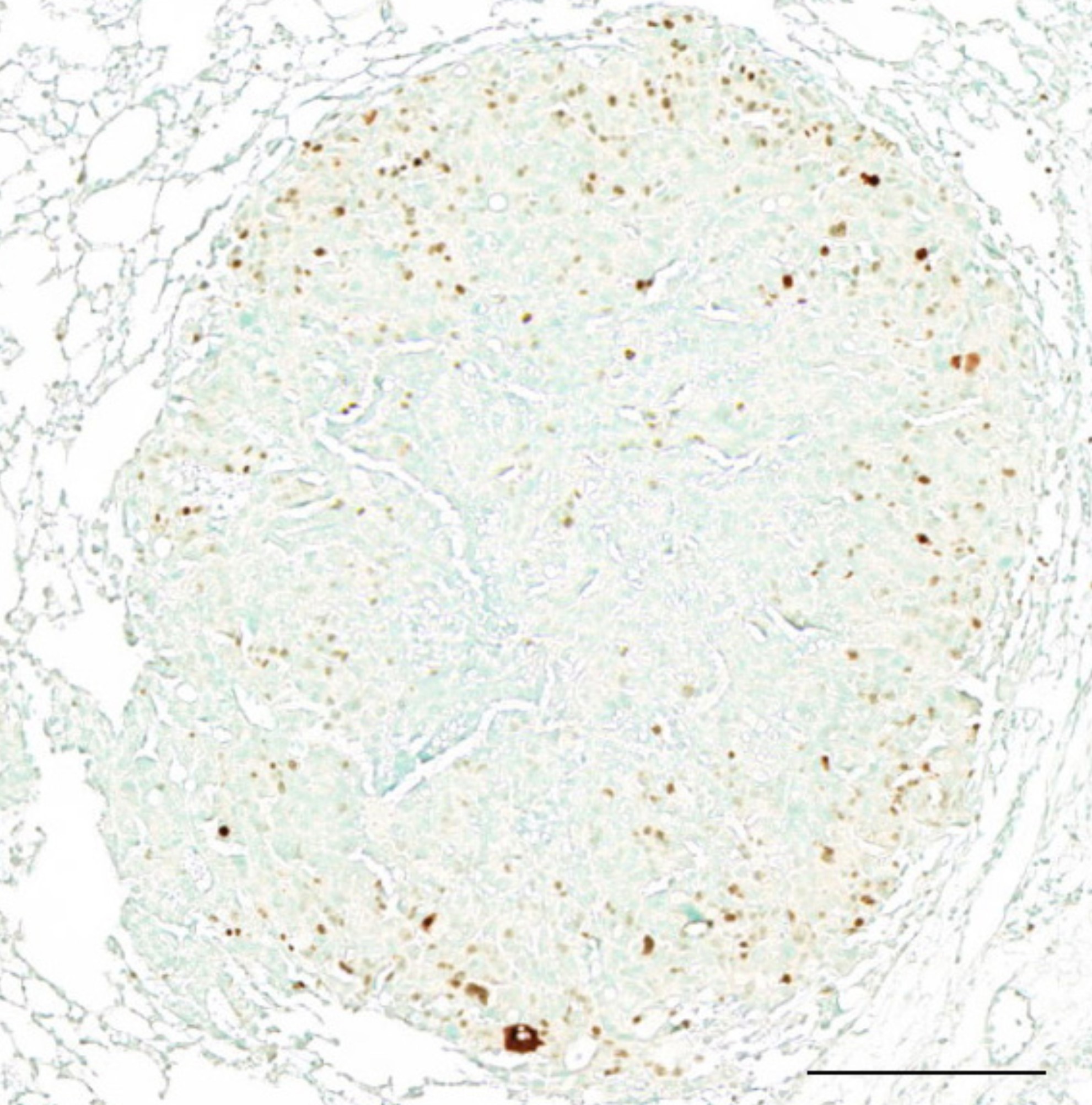

Supplement: Supplementary file 4 — Source data Fig. 3 [file 44321_2024_138_MOESM4_ESM.zip › Figure 3/3C/Seliciclib.jpg]

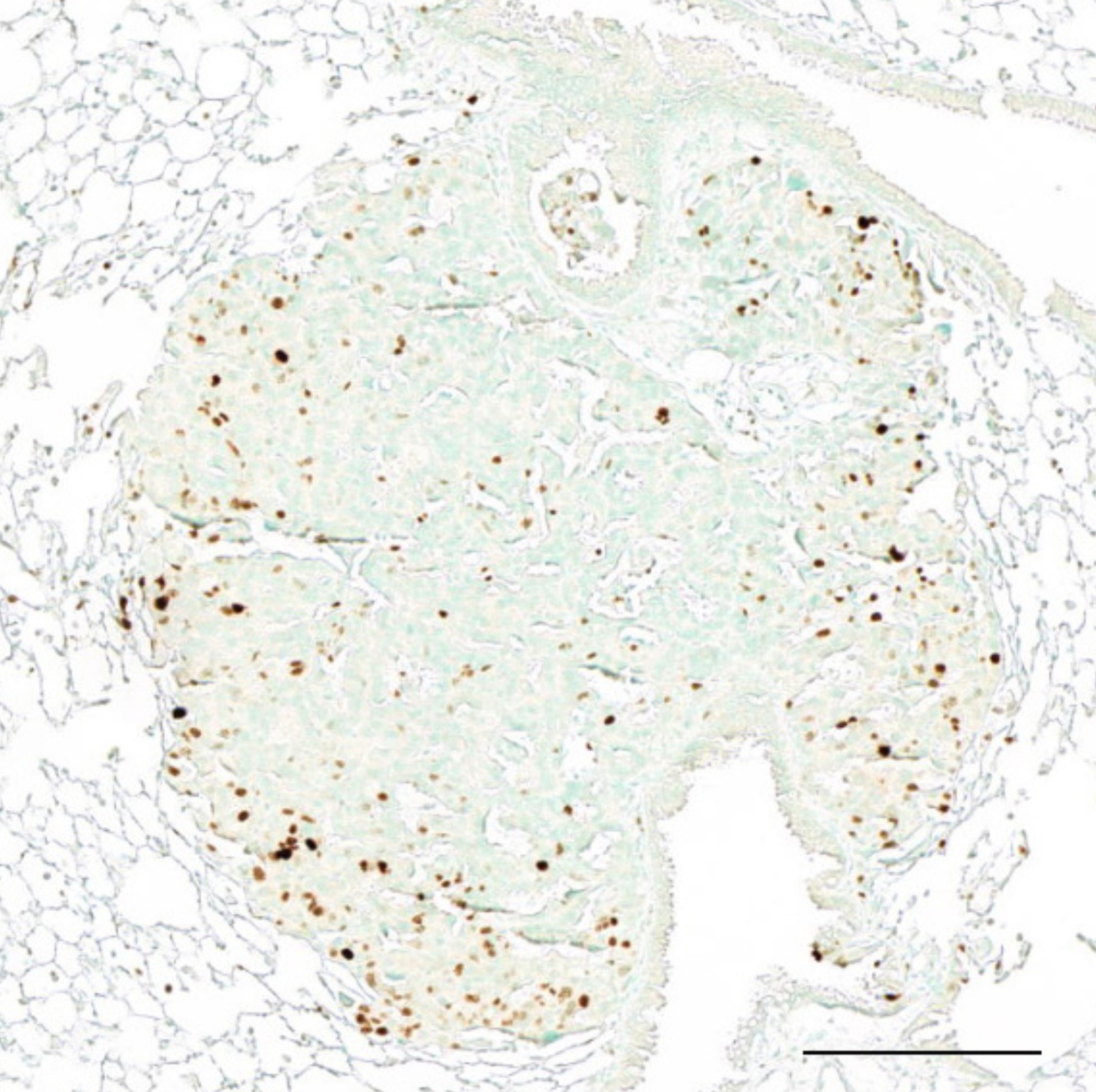

Supplement: Supplementary file 4 — Source data Fig. 3 [file 44321_2024_138_MOESM4_ESM.zip › Figure 3/3C/XMD8-92.jpg]

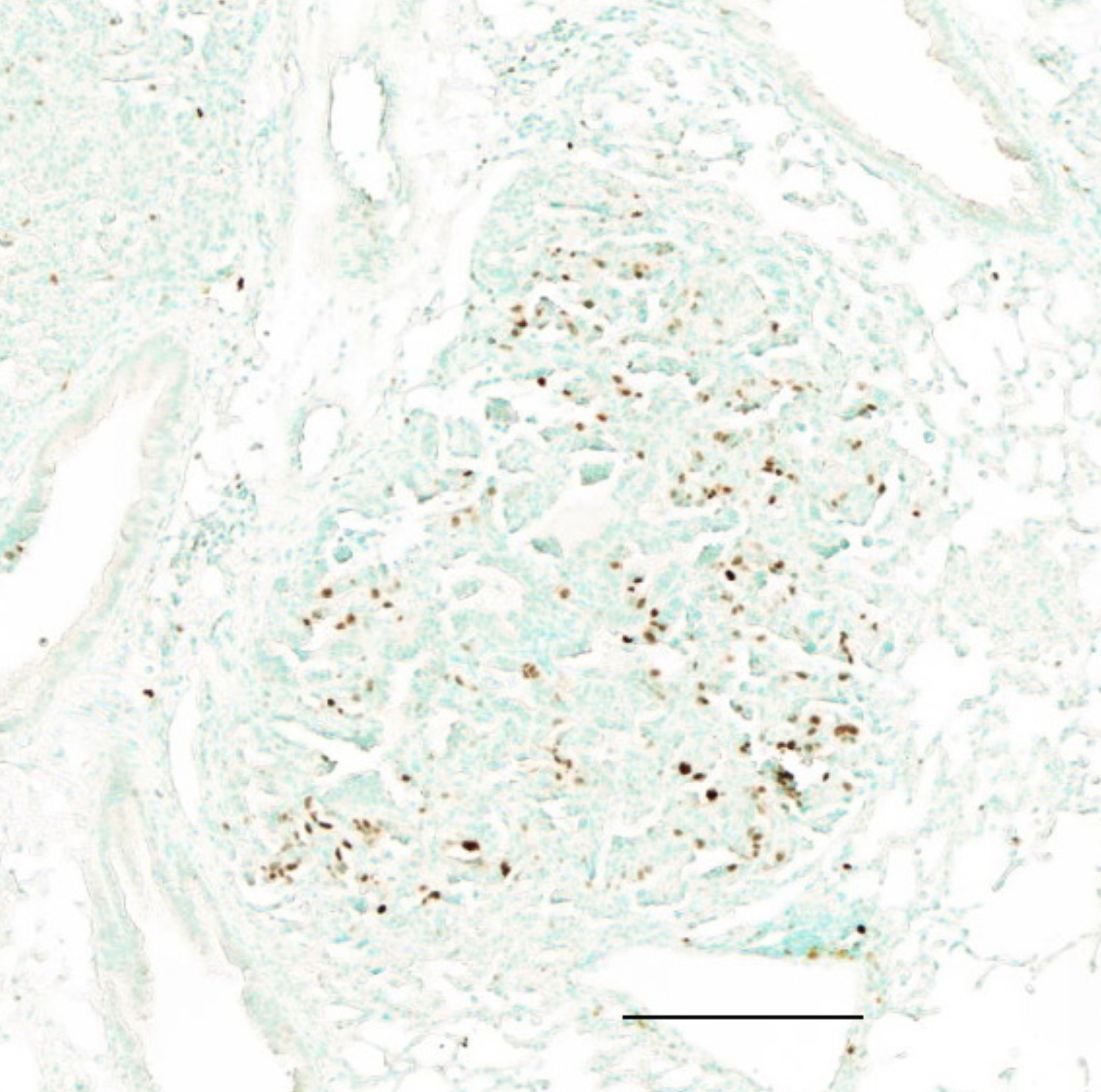

Supplement: Supplementary file 4 — Source data Fig. 3 [file 44321_2024_138_MOESM4_ESM.zip › Figure 3/3C/Vehicle.jpg]

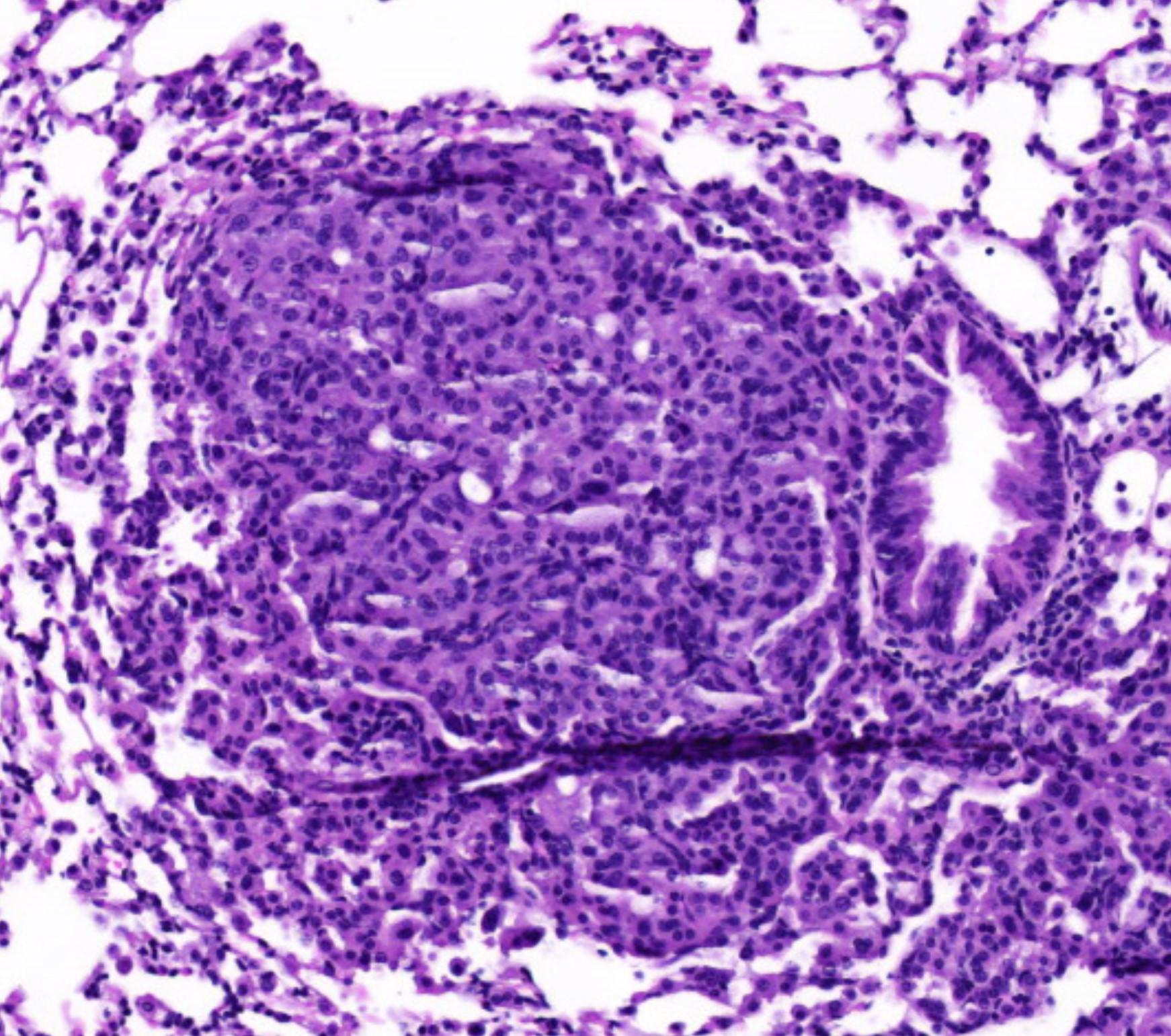

Supplement: Supplementary file 4 — Source data Fig. 3 [file 44321_2024_138_MOESM4_ESM.zip › Figure 3/3B/Fig. 3B- H&E images/Seliciclib zoom.jpg]

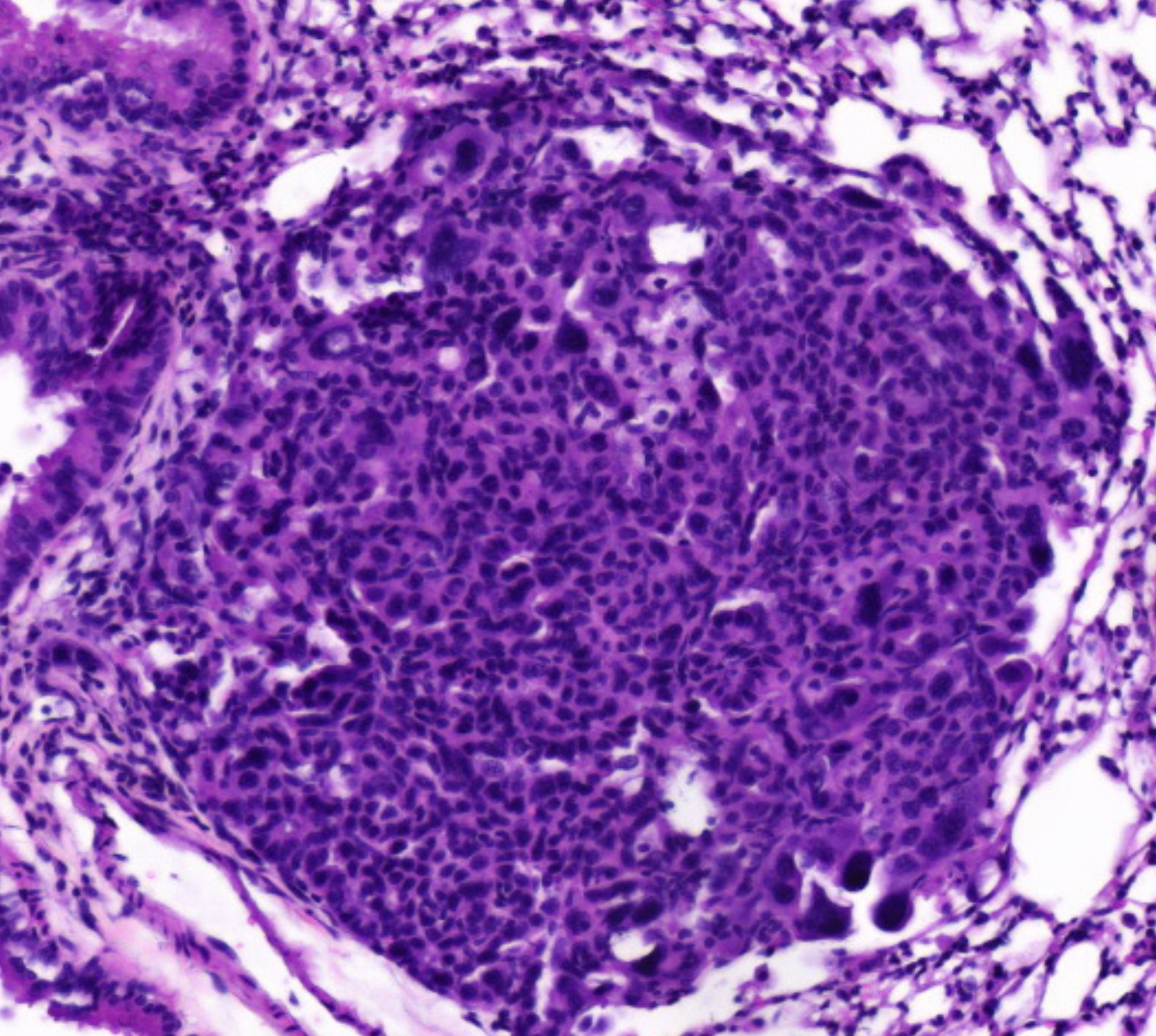

Supplement: Supplementary file 4 — Source data Fig. 3 [file 44321_2024_138_MOESM4_ESM.zip › Figure 3/3B/Fig. 3B- H&E images/Combination zoom.jpg]

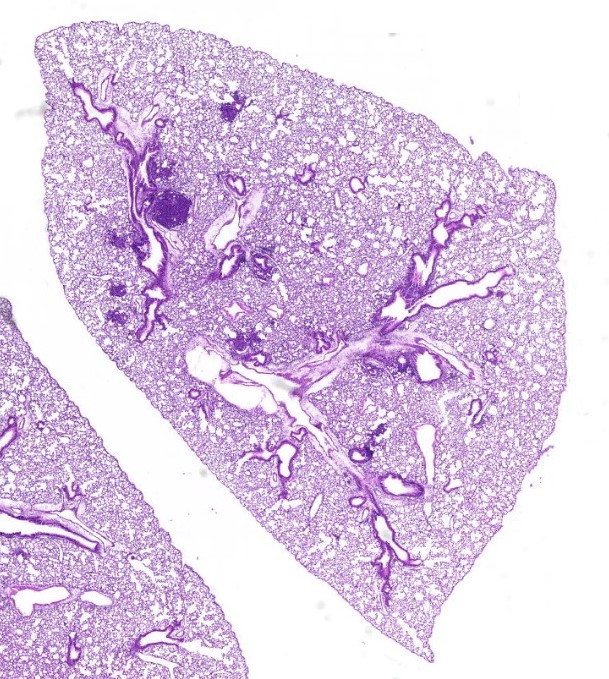

Supplement: Supplementary file 4 — Source data Fig. 3 [file 44321_2024_138_MOESM4_ESM.zip › Figure 3/3B/Fig. 3B- H&E images/Combination.jpg]

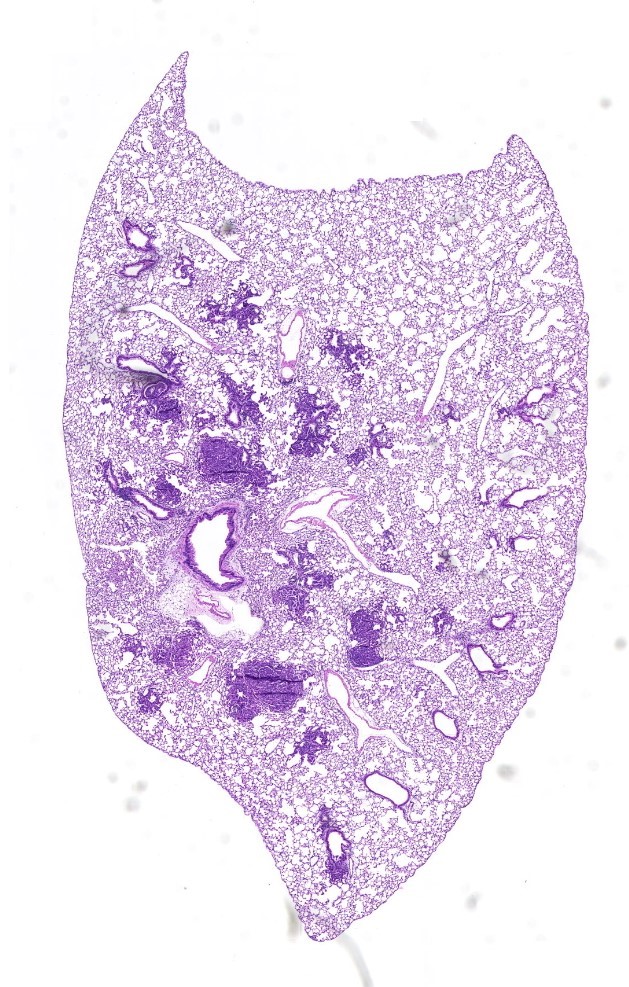

Supplement: Supplementary file 4 — Source data Fig. 3 [file 44321_2024_138_MOESM4_ESM.zip › Figure 3/3B/Fig. 3B- H&E images/Seliciclib.jpg]

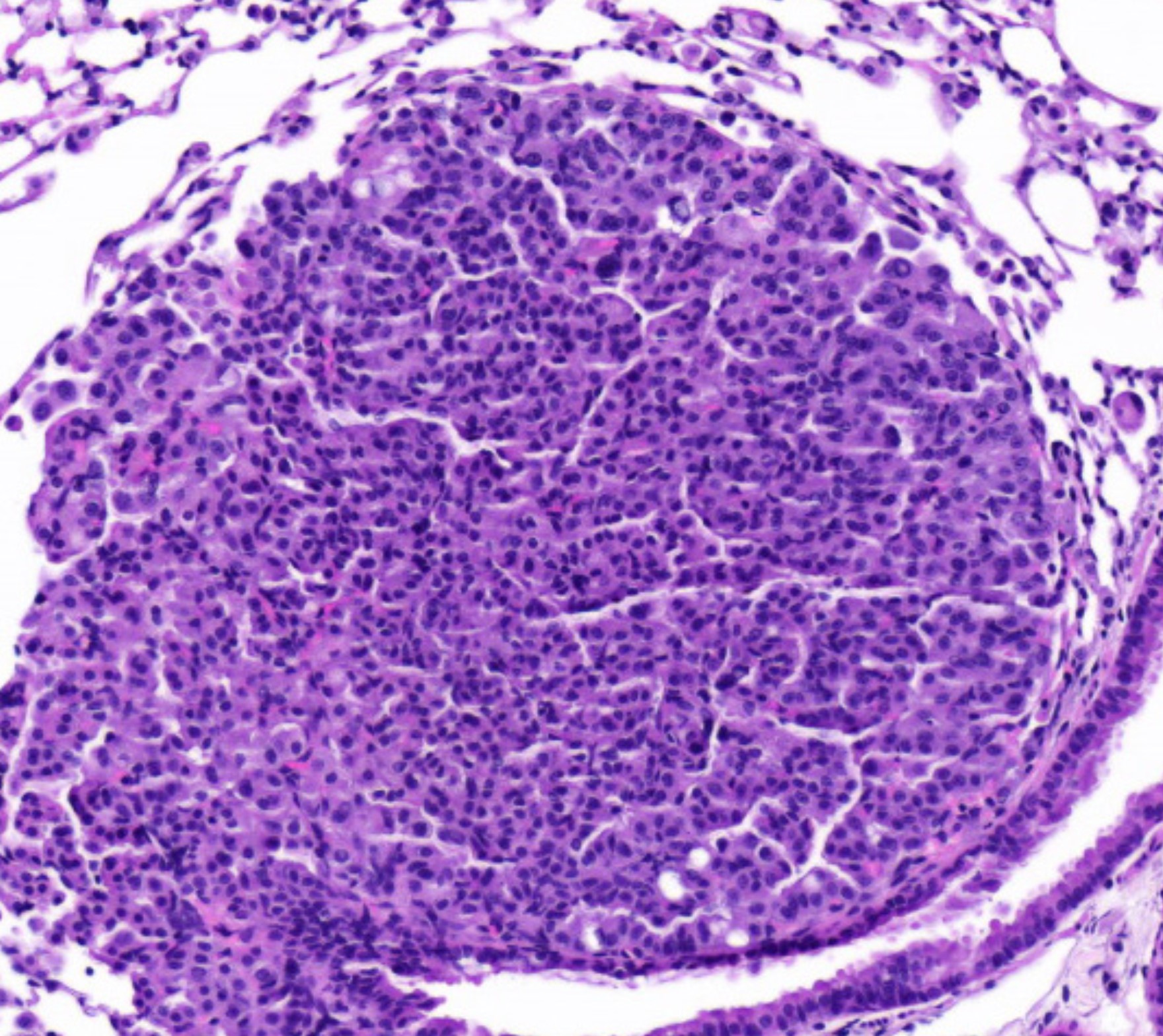

Supplement: Supplementary file 4 — Source data Fig. 3 [file 44321_2024_138_MOESM4_ESM.zip › Figure 3/3B/Fig. 3B- H&E images/Vehicle zoom.jpg]

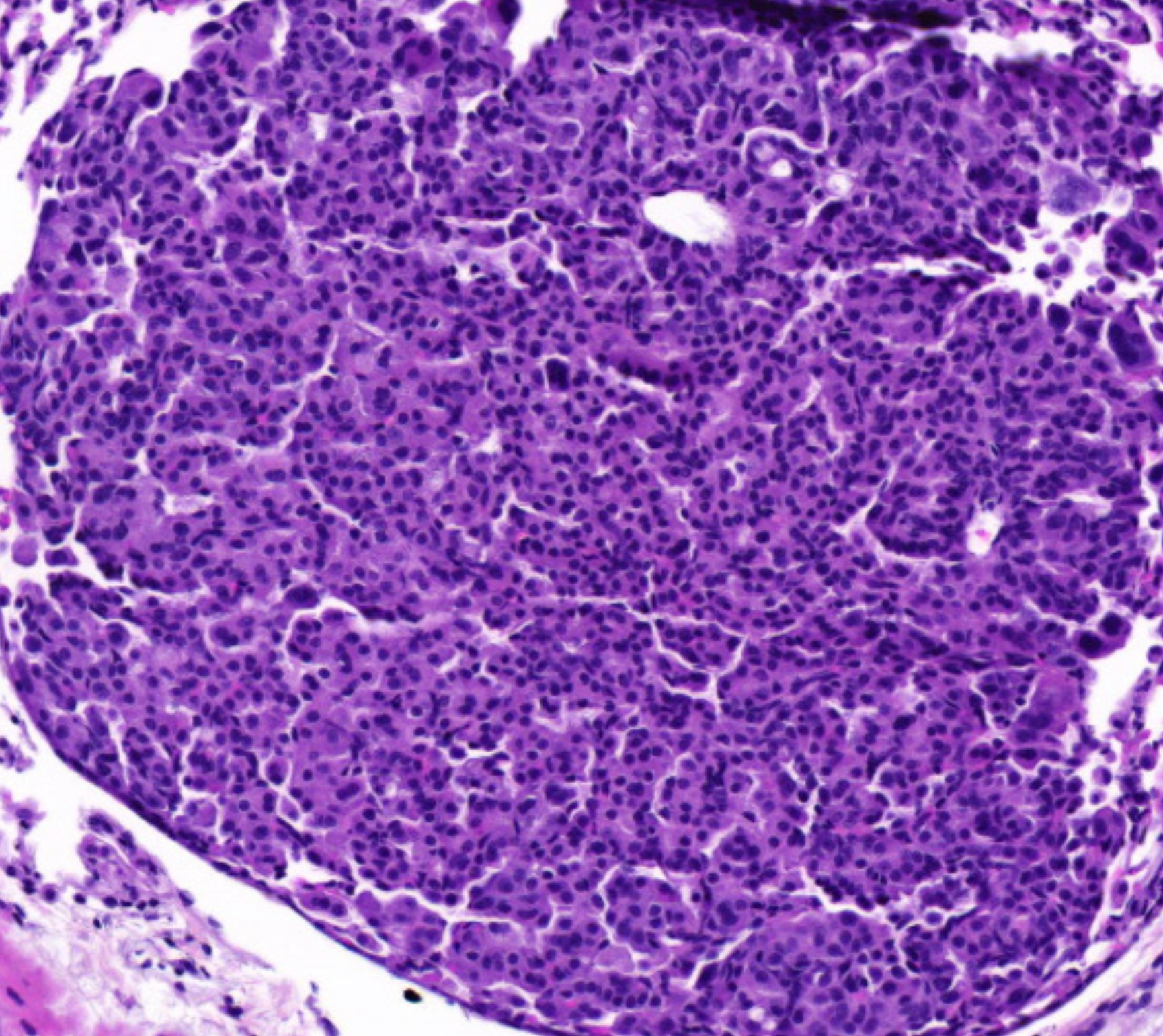

Supplement: Supplementary file 4 — Source data Fig. 3 [file 44321_2024_138_MOESM4_ESM.zip › Figure 3/3B/Fig. 3B- H&E images/XMD8-92 zoom.jpg]

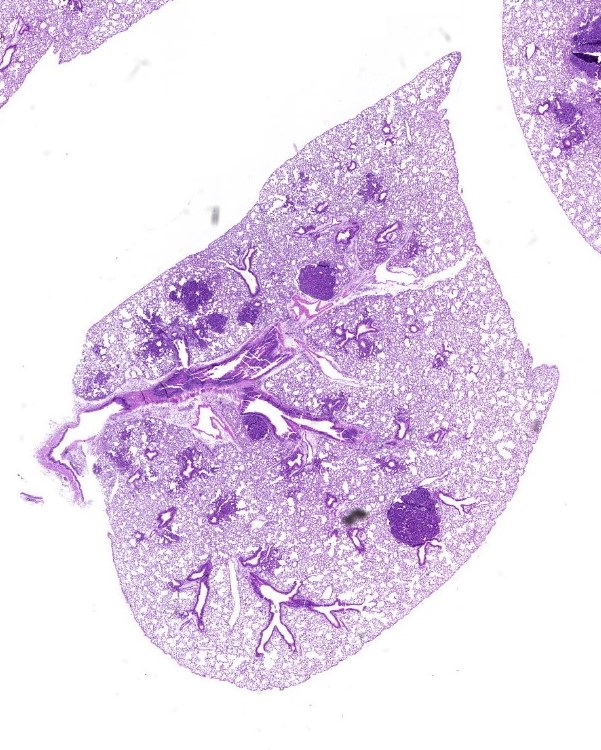

Supplement: Supplementary file 4 — Source data Fig. 3 [file 44321_2024_138_MOESM4_ESM.zip › Figure 3/3B/Fig. 3B- H&E images/XMD8-92.jpg]

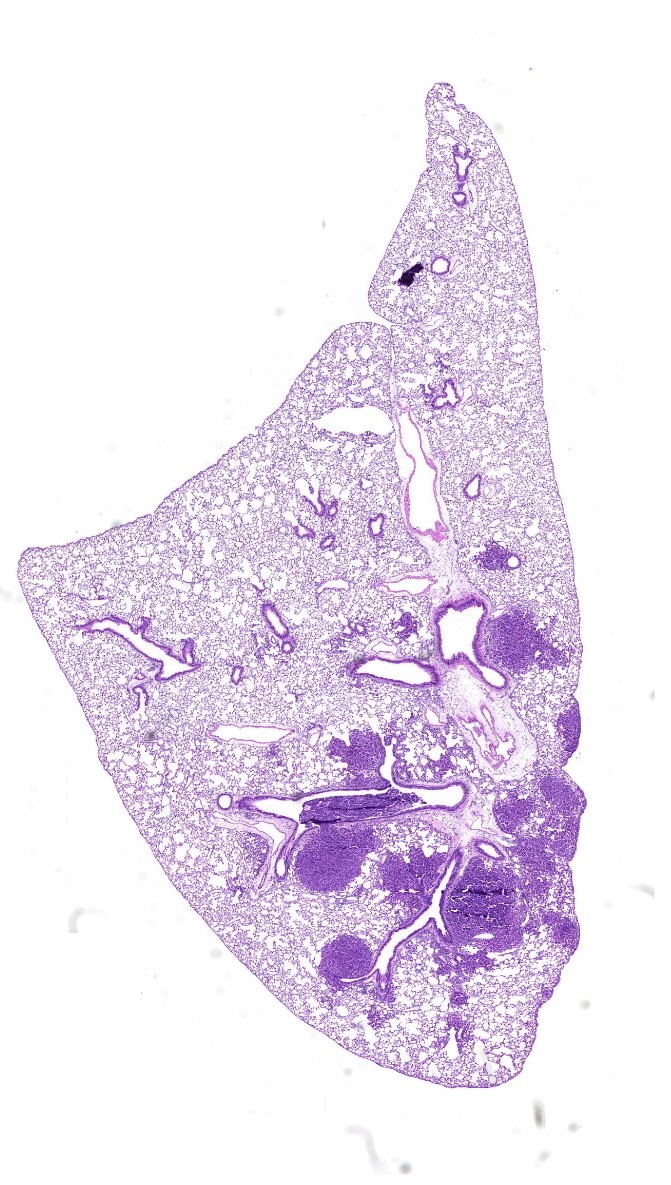

Supplement: Supplementary file 4 — Source data Fig. 3 [file 44321_2024_138_MOESM4_ESM.zip › Figure 3/3B/Fig. 3B- H&E images/Vehicle.jpg]

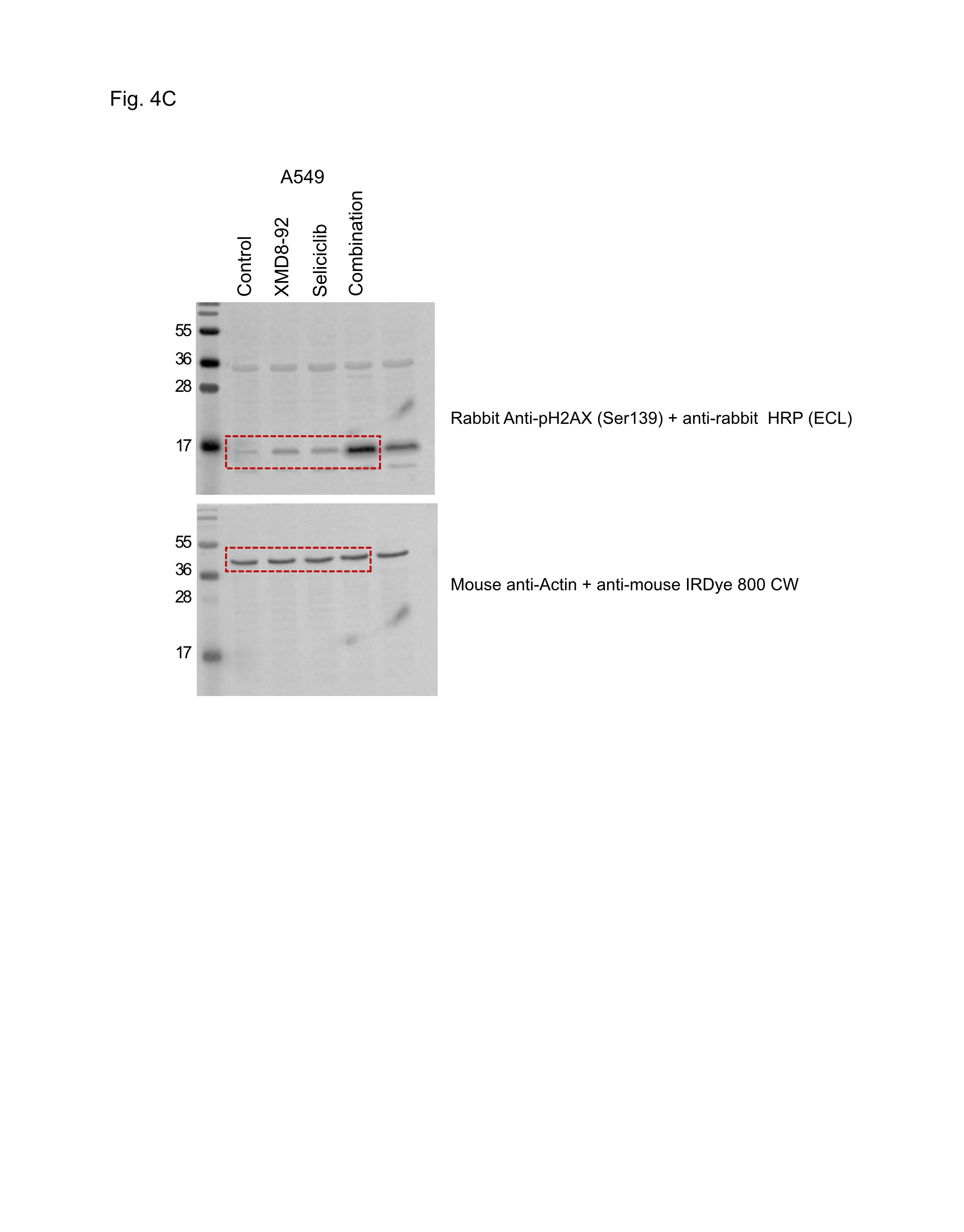

Supplement: Supplementary file 5 — Source data Fig. 4 [file 44321_2024_138_MOESM5_ESM.zip › Figure 4/4C/Fig. 4C-immunoblot.tiff]

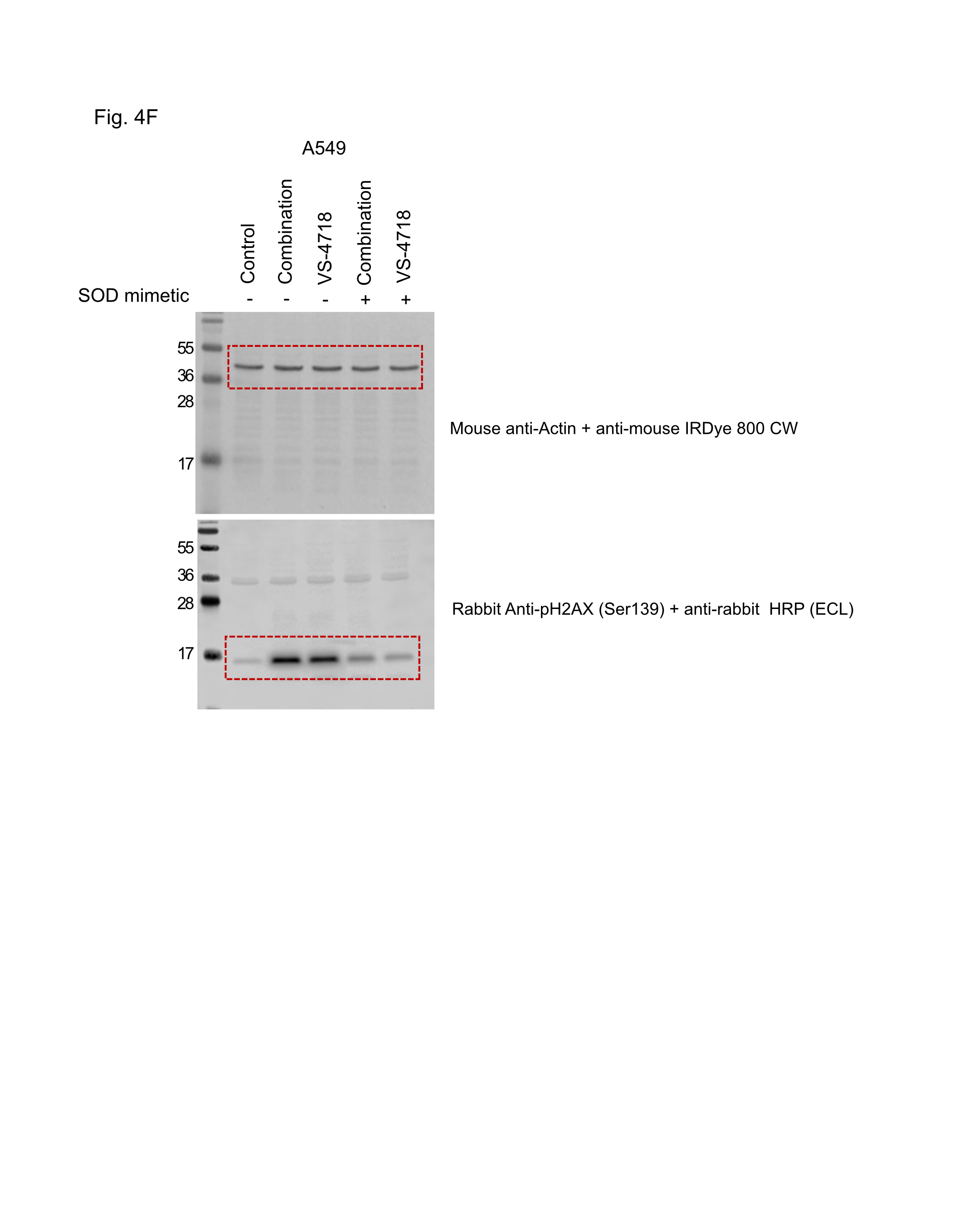

Supplement: Supplementary file 5 — Source data Fig. 4 [file 44321_2024_138_MOESM5_ESM.zip › Figure 4/4F/Fig. 4F-immunoblot.tiff]

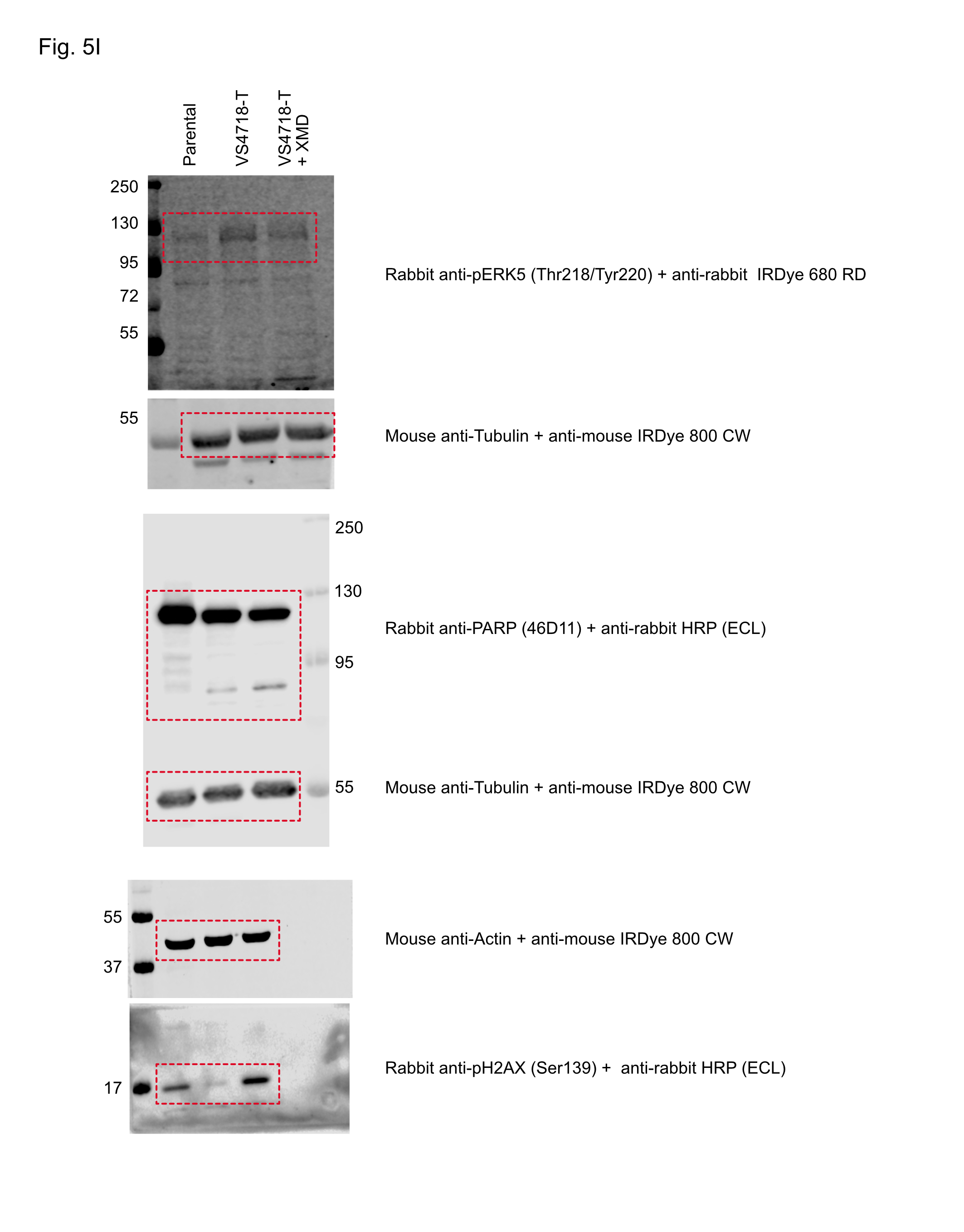

Supplement: Supplementary file 6 — Source data Fig. 5 [file 44321_2024_138_MOESM6_ESM.zip › Figure 5/5I/Fig. 5I-immunoblot.tiff]

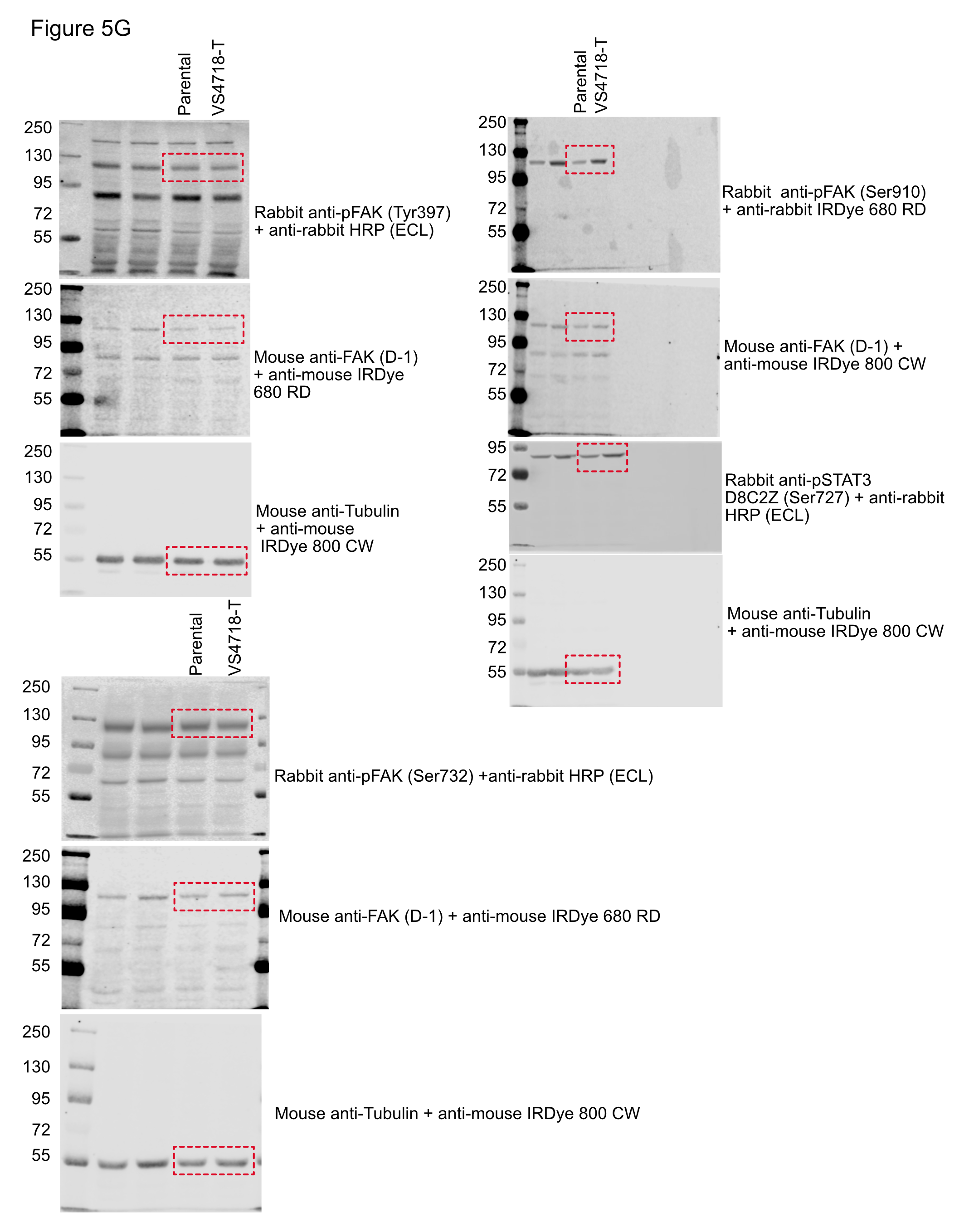

Supplement: Supplementary file 6 — Source data Fig. 5 [file 44321_2024_138_MOESM6_ESM.zip › Figure 5/5G/Fig. 5G-Immunoblot.tiff]

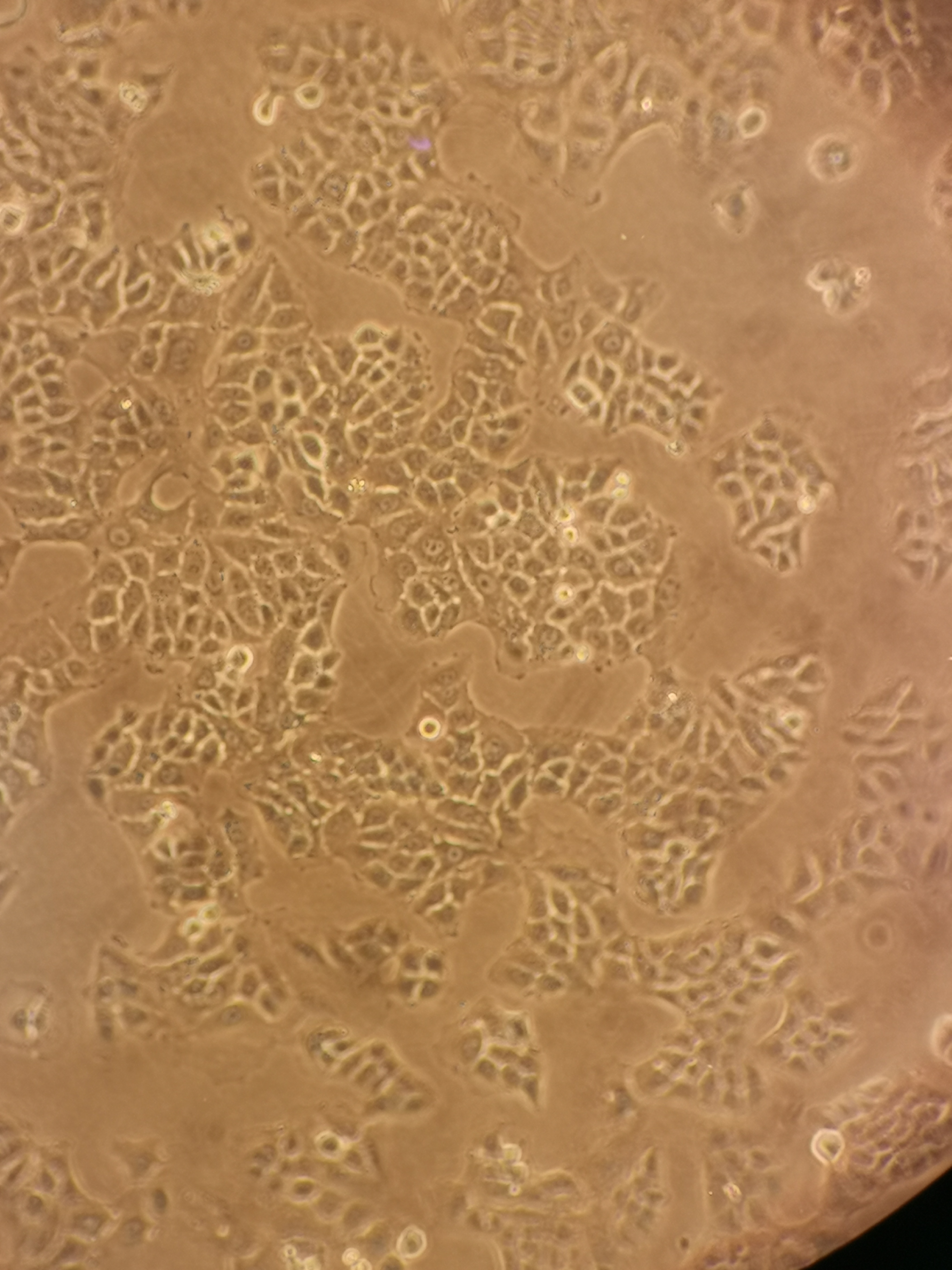

Supplement: Supplementary file 6 — Source data Fig. 5 [file 44321_2024_138_MOESM6_ESM.zip › Figure 5/5A/Parental images cells.jpg]

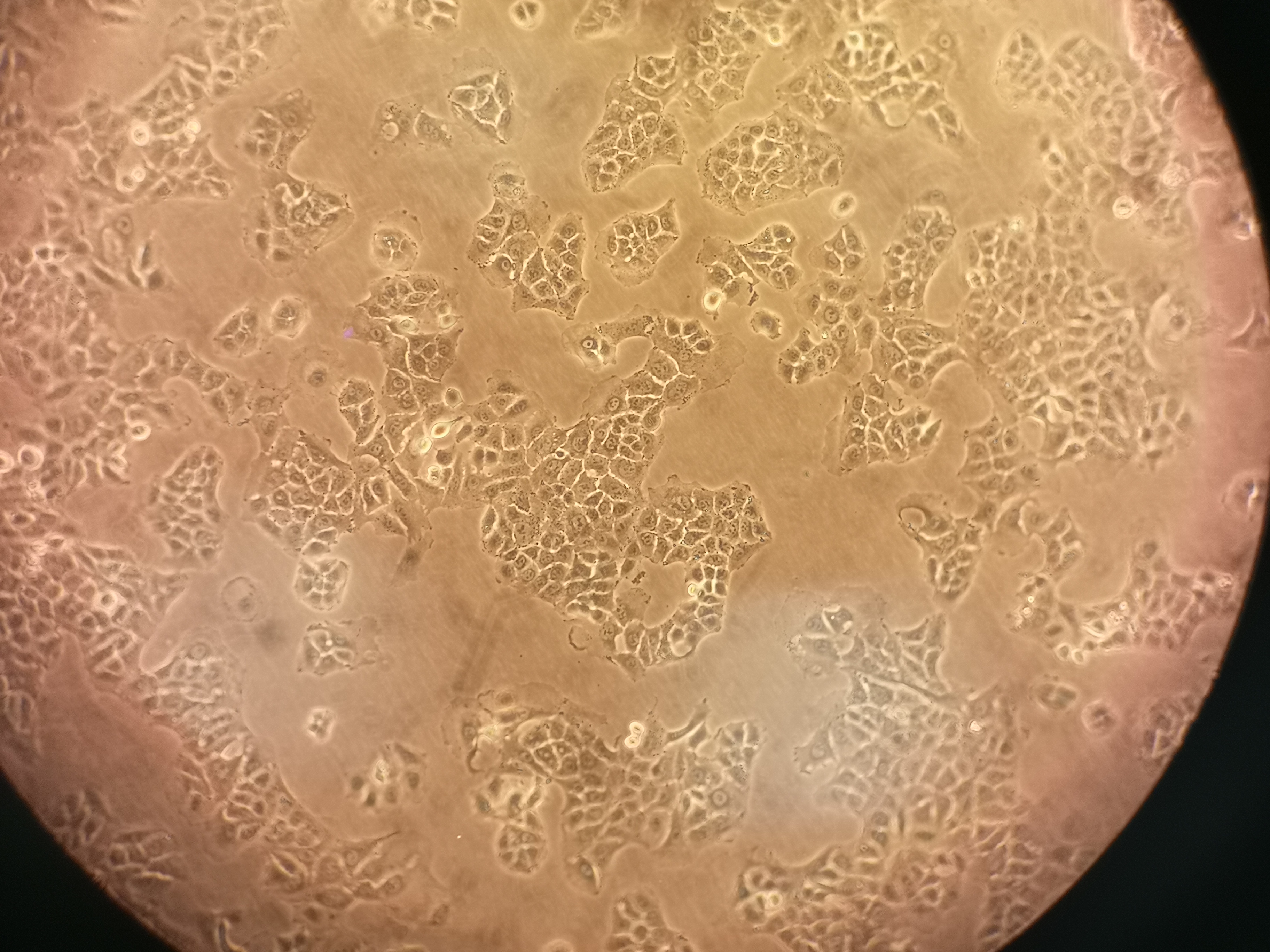

Supplement: Supplementary file 6 — Source data Fig. 5 [file 44321_2024_138_MOESM6_ESM.zip › Figure 5/5A/VS4718-T removal_cells.jpg]

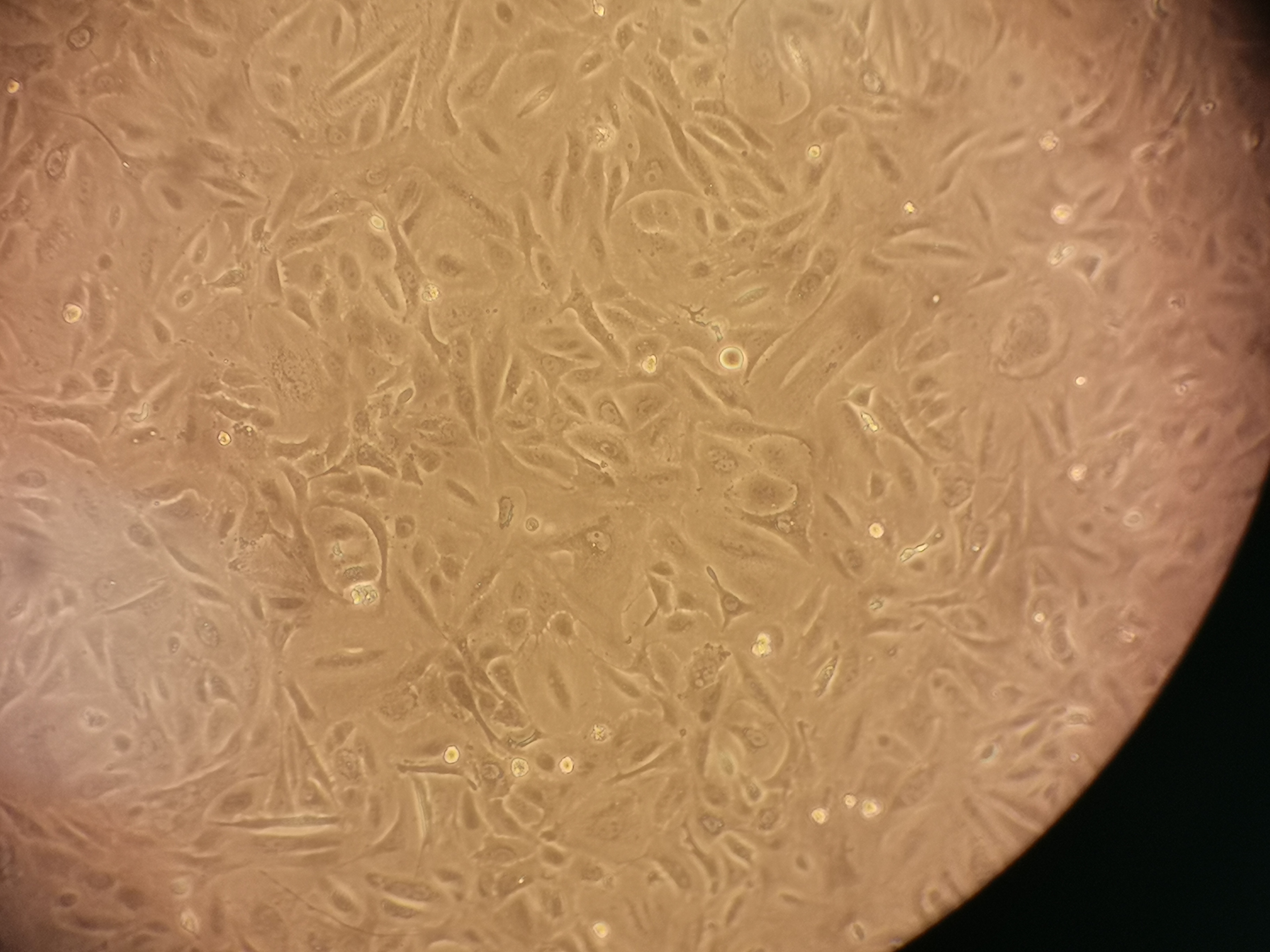

Supplement: Supplementary file 6 — Source data Fig. 5 [file 44321_2024_138_MOESM6_ESM.zip › Figure 5/5A/VS4718-T_images cells.jpg]

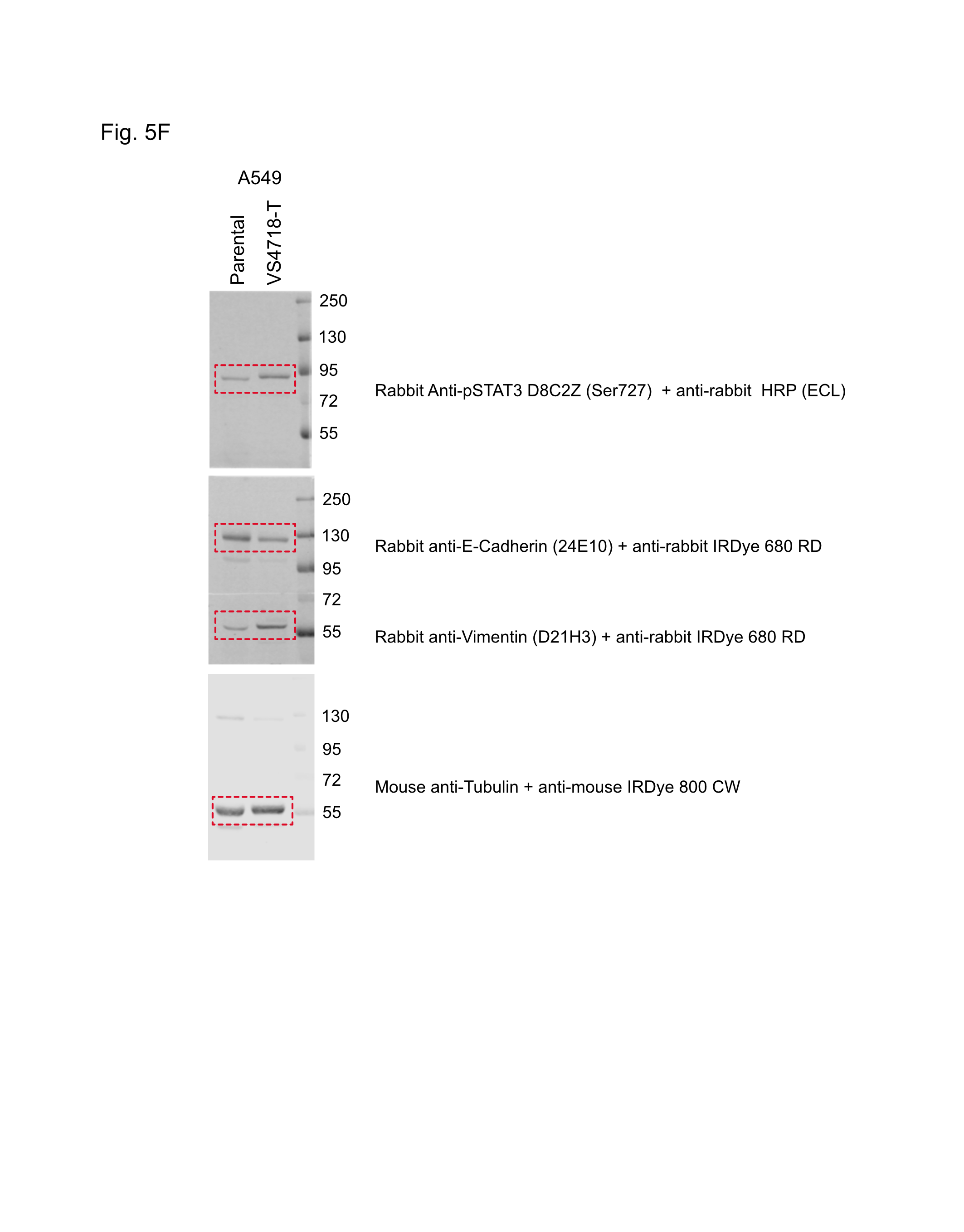

Supplement: Supplementary file 6 — Source data Fig. 5 [file 44321_2024_138_MOESM6_ESM.zip › Figure 5/5F/Fig. 5F-immunoblot.tiff]

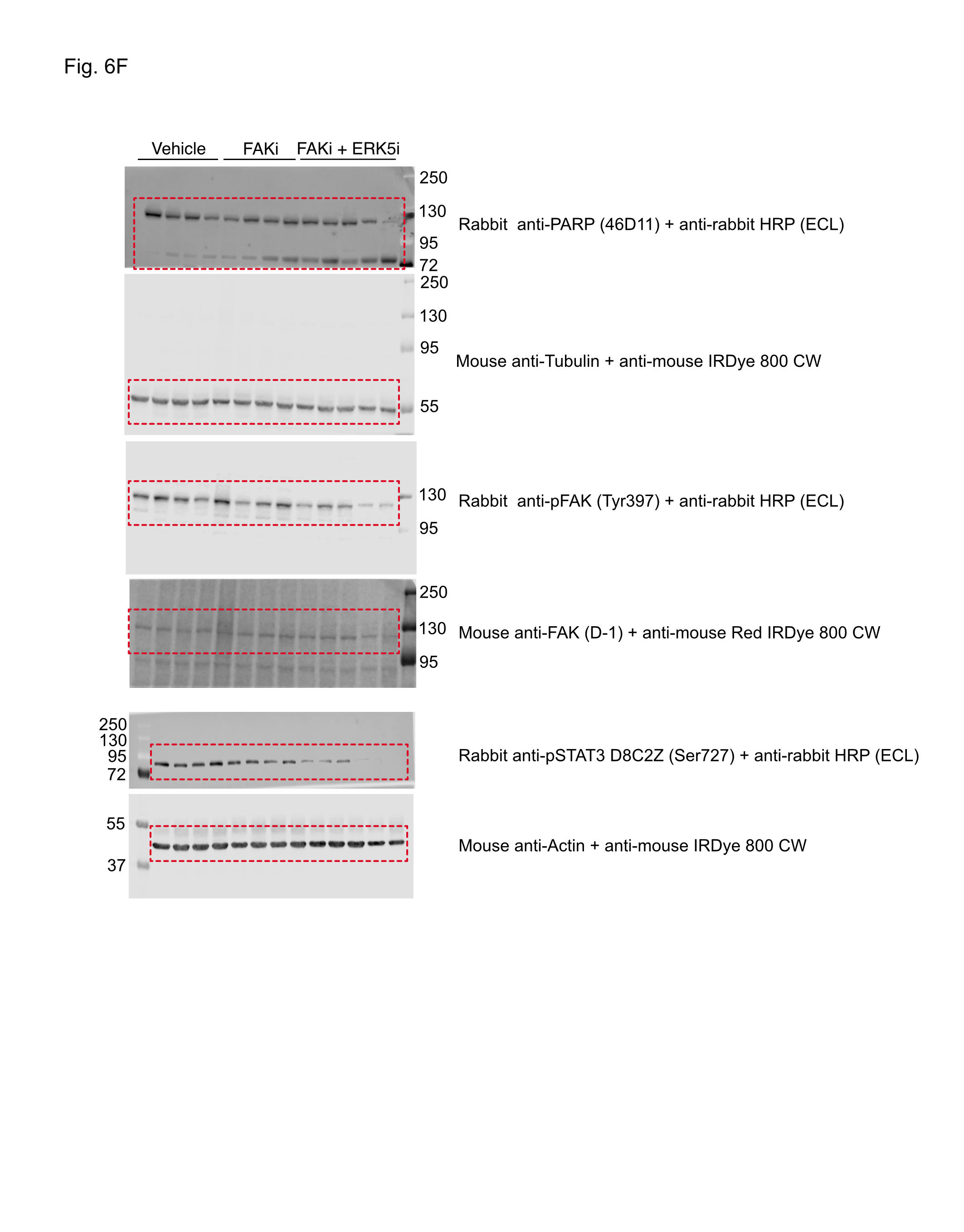

Supplement: Supplementary file 7 — Source data Fig. 6 [file 44321_2024_138_MOESM7_ESM.zip › Figure 6/6F/Fig. 6F-immunoblot.tiff]

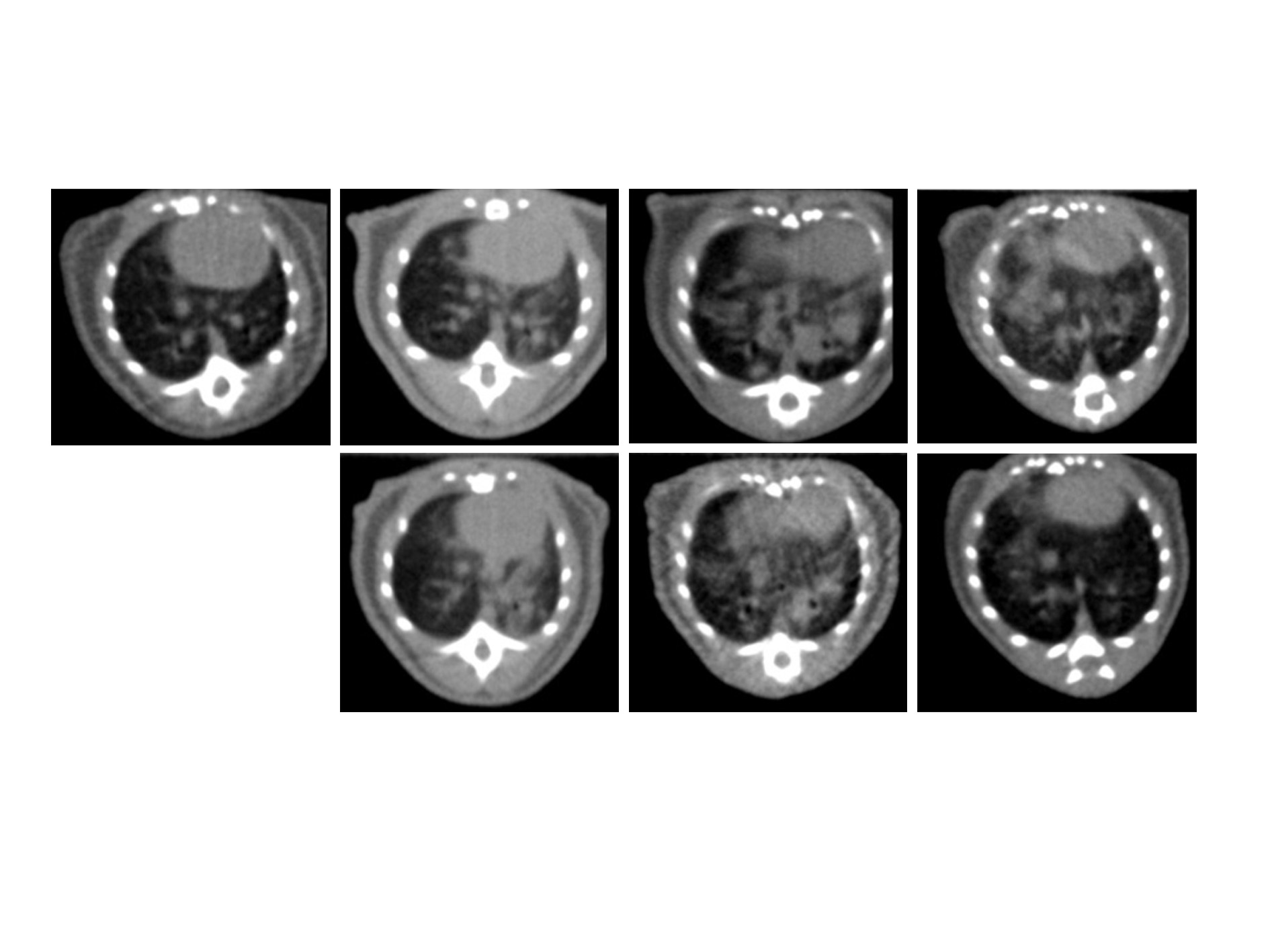

Supplement: Supplementary file 7 — Source data Fig. 6 [file 44321_2024_138_MOESM7_ESM.zip › Figure 6/6B/CT scan images full resolution.jpg]

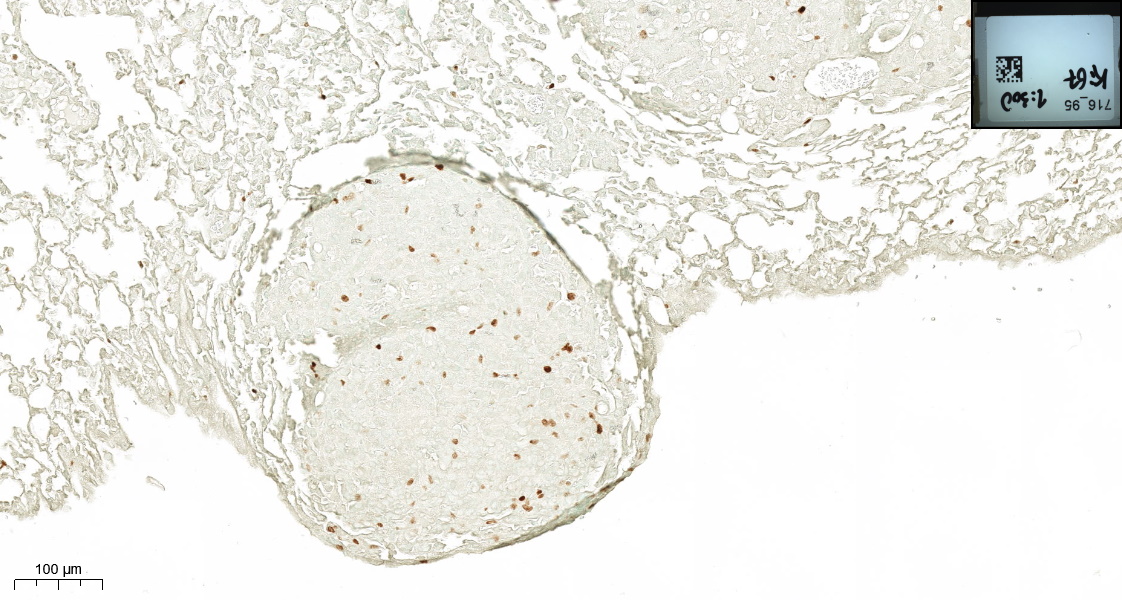

Supplement: Supplementary file 7 — Source data Fig. 6 [file 44321_2024_138_MOESM7_ESM.zip › Figure 6/6E/FAKi_Ki67_20.0x.jpg]

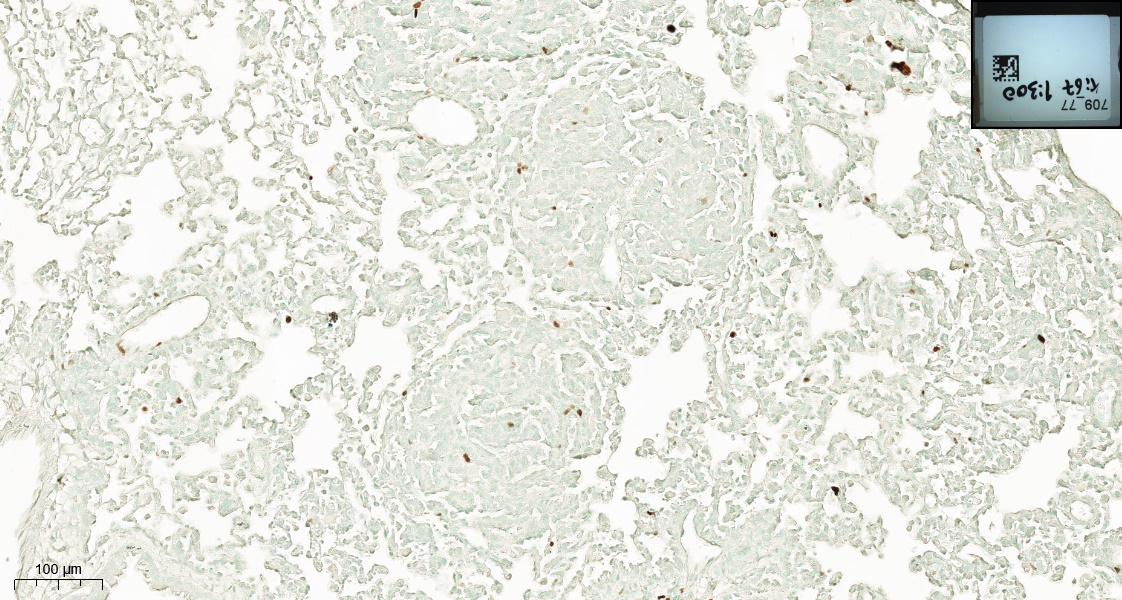

Supplement: Supplementary file 7 — Source data Fig. 6 [file 44321_2024_138_MOESM7_ESM.zip › Figure 6/6E/FAKi and ERK5i_Ki67_20.0x.jpg]

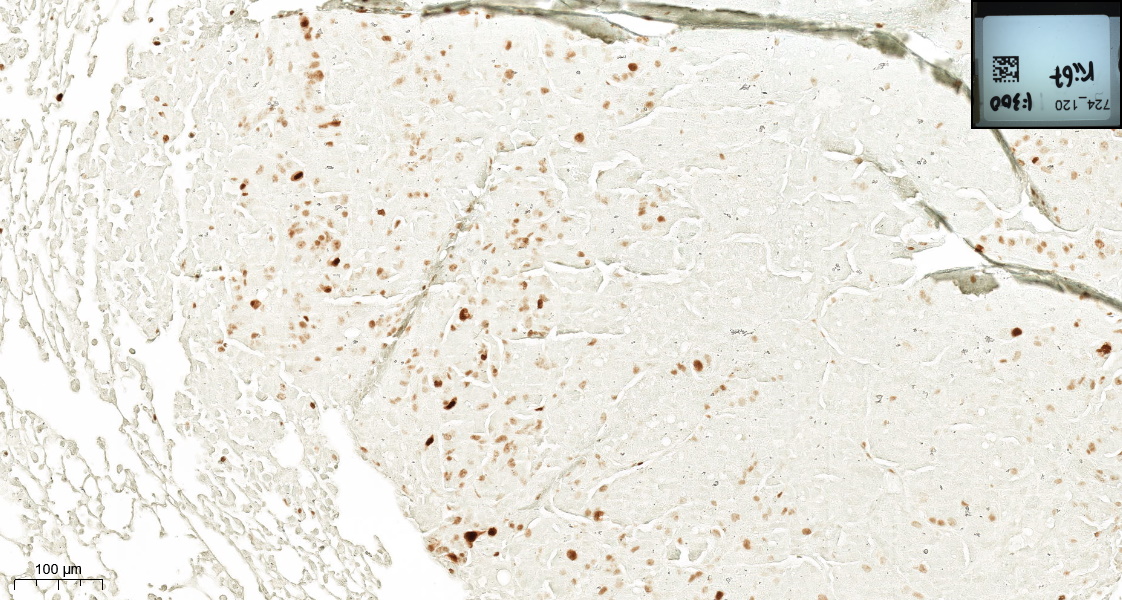

Supplement: Supplementary file 7 — Source data Fig. 6 [file 44321_2024_138_MOESM7_ESM.zip › Figure 6/6E/Vehicle_Ki67_20.0x_1.jpg]

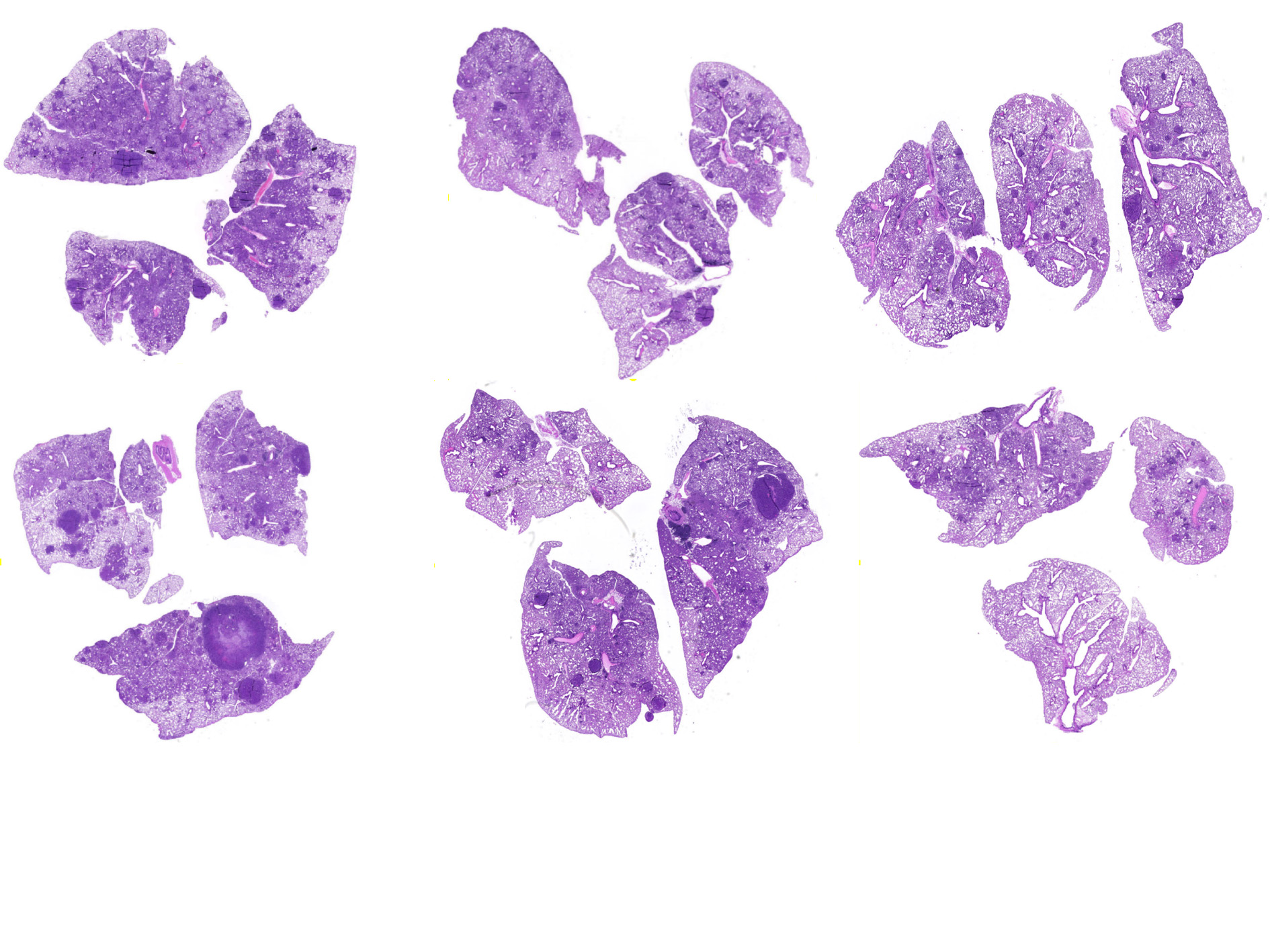

Supplement: Supplementary file 7 — Source data Fig. 6 [file 44321_2024_138_MOESM7_ESM.zip › Figure 6/6D/Fig. 6D-H and E images.jpg]
