# Supplementary material for: ERK5 suppression overcomes FAK inhibitor resistance in mutant KRAS-driven non-small cell lung cancer
Source: EMBO Mol Med. 2024 Sep 13;16(10):2402–26. doi: 10.1038/s44321-024-00138-7 (PMC11473843; doi:10.1038/s44321-024-00138-7)
Supplement: Supplementary file 8 — Expanded View Figures [file 44321_2024_138_MOESM8_ESM.pdf]

Expanded View Figures

Figure EV1. Functional characterization of the different human FAK phospho-mutants.

(**A**) Immunoblot analysis of the indicated targets of HBEC3-FAK KO cell line transduced with a lentiviral plasmid carrying either a control shRNA (scramble) or a shRNA against human p53. (**B**) Immunoblot analysis of the indicated targets in parental and FAK knockout HBEC3 cell line transduced with either an empty vector or a plasmid encoding mutant KRAS (left) and relative cell number of the indicated groups calculated at day 4 (right); $n = 3$. MutKRAS: mutant KRAS; ns: not significant. (**C**) Relative cell number of HBEC3-FAK KO cell line transduced either with empty vector (pWZL-Hygro) or with the FAK phospho-mutant Y397F (pWZL-Hygro-FAK Y397F) in the absence or presence of mutKRAS (pBABE-zeo or pBABE-zeo-KRAS ^{G12D}). MutKRAS: mutant KRAS; $n = 3$. Note that the Empty vector and Empty vector + mutKRAS group plots are the same as in main Fig. 1E because these experiments were performed at the same time for direct comparison. (**D**) Relative quantification of colony forming capacity of HBEC3-FAK KO cells previously transduced as indicated; $n = 3$. (**E**) Immunoblot analysis of the indicated targets in HBEC3-FAK KO cells transduced either with empty vector (pWZL-Hygro) or with the FAK phospho-mutant Y397F (pWZL-Hygro-FAK Y397F). (**F**) Immunoblot analysis of the indicated targets in HBEC3-FAK KO transduced either with empty vector (pWZL-Hygro) or wild-type FAK (pWZL Hygro-FAK WT) or with the indicated human FAK phospho-mutants. (**G–J**) Relative cell number of HBEC3-FAK KO cell line transduced either with wild-type FAK (pWZL Hygro-FAK WT) or the indicated human FAK phospho-mutants in the absence or presence of mutKRAS^{G12D} (pBABE-zeo or pBABE-zeo-KRAS ^{G12D}). MutKRAS: mutant KRAS; $n = 3$. Graphical data are mean ± SD. Statistical analyses were done using two-tailed unpaired Student's *t* test or one-way ANOVA; *n*, number of biologically independent samples.

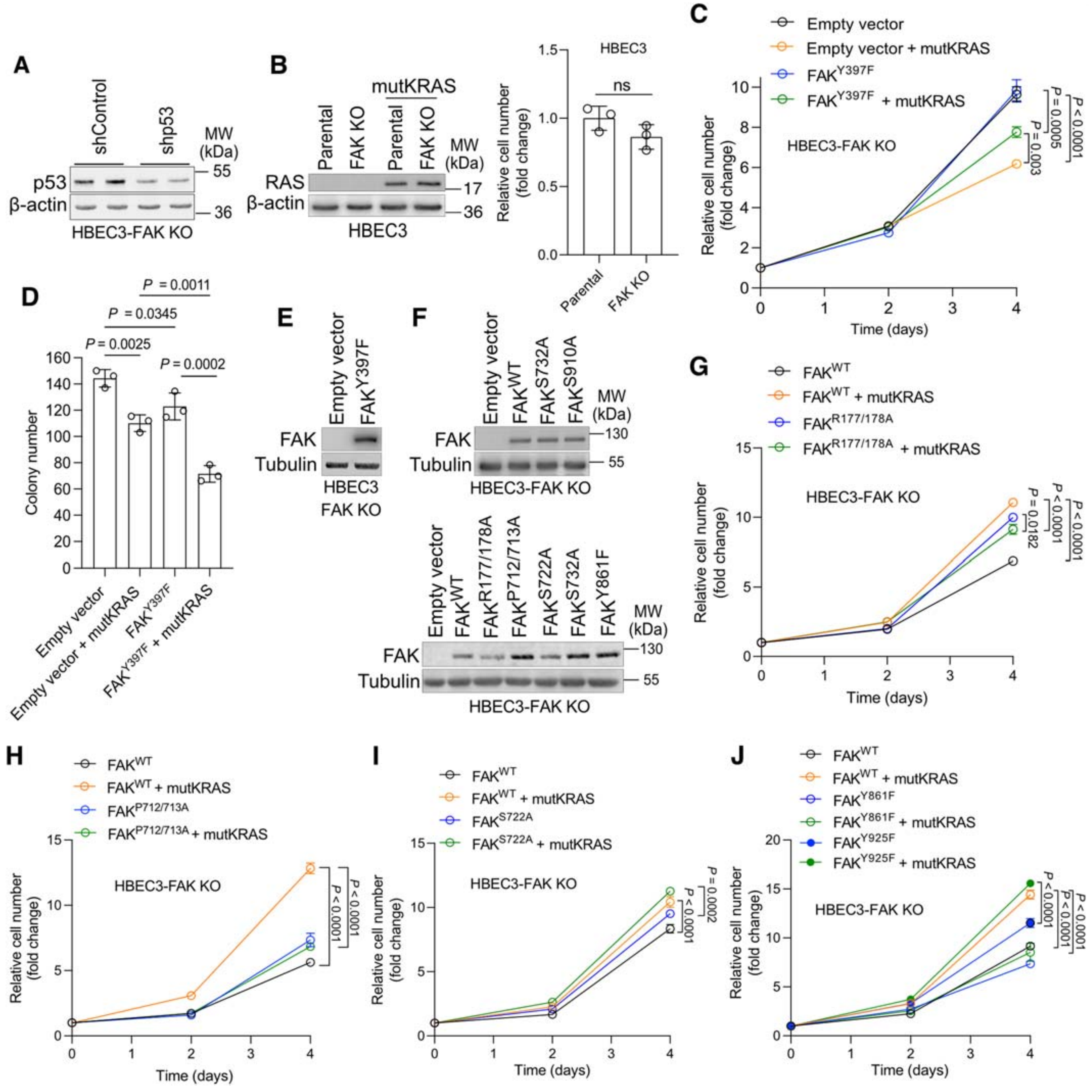
A
shControl
shp53
MW (kDa)
p53
β-actin
55
36
HBEC3-FAK KO
B
mutKRAS
Parental
FAK KO
Parental
FAK KO
MW (kDa)
RAS
β-actin
17
36
HBEC3
HBEC3
ns
Relative cell number (fold change)
Parental
FAK KO
C
Empty vector
Empty vector + mutKRAS
FAKY397F
FAKY397F + mutKRAS
HBEC3-FAK KO
P < 0.0001
P = 0.0005
P = 0.003
Relative cell number (fold change)
Time (days)
D
P = 0.0011
P = 0.0345
P = 0.0025
P = 0.0002
Colony number
Empty vector
Empty vector + mutKRAS
FAKY397F
FAKY397F + mutKRAS
E
Empty vector
FAKY397F
FAK
Tubulin
HBEC3 FAK KO
F
Empty vector
FAKWT
FAKS732A
FAKS910A
MW (kDa)
FAK
Tubulin
130
55
HBEC3-FAK KO
Empty vector
FAKWT
FAKR177/178A
FAKP712/713A
FAKS722A
FAKS732A
FAKY861F
MW (kDa)
FAK
Tubulin
130
55
HBEC3-FAK KO
G
FAKWT
FAKWT + mutKRAS
FAKR177/178A
FAKR177/178A + mutKRAS
HBEC3-FAK KO
P = 0.0182
P < 0.0001
P < 0.0001
Relative cell number (fold change)
Time (days)
H
FAKWT
FAKWT + mutKRAS
FAKP712/713A
FAKP712/713A + mutKRAS
HBEC3-FAK KO
P < 0.0001
P < 0.0001
Relative cell number (fold change)
Time (days)
I
FAKWT
FAKWT + mutKRAS
FAKS722A
FAKS722A + mutKRAS
HBEC3-FAK KO
P = 0.0002
P < 0.0001
Relative cell number (fold change)
Time (days)
J
FAKWT
FAKWT + mutKRAS
FAKY861F
FAKY861F + mutKRAS
FAKY925F
FAKY925F + mutKRAS
HBEC3-FAK KO
P < 0.0001
P < 0.0001
P < 0.0001
Relative cell number (fold change)
Time (days)

 © The Author(s)

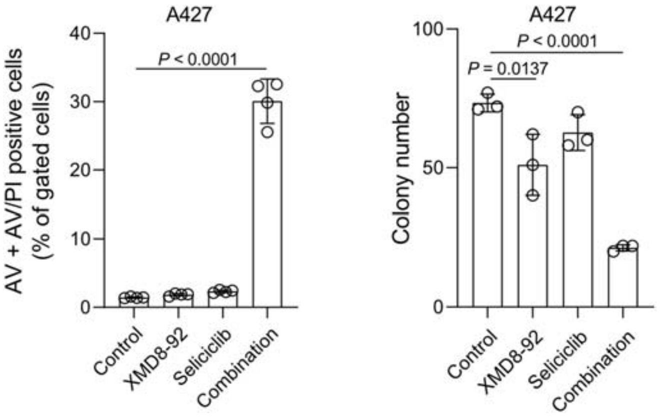

Figure EV2. Co-inhibition of ERK5 and CDK5 increases apoptosis and suppresses colony forming capacity of KRAS mutant NSCLC cells.

Relative quantification of cell death by flow cytometry analysis of Annexin V-Atto 633 (AV) + Annexin V/PI (AV/PI)-positive (left) and colony number (right) of A427 cells treated with XMD8-92 or Seliciclib (10 µM for apoptosis assay and 2.5 µM for colony formation each, respectively) alone or in combination; $n = 3$. Graphical data are mean ± SD. Statistical analyses were done using one-way ANOVA; n, number of biologically independent samples.

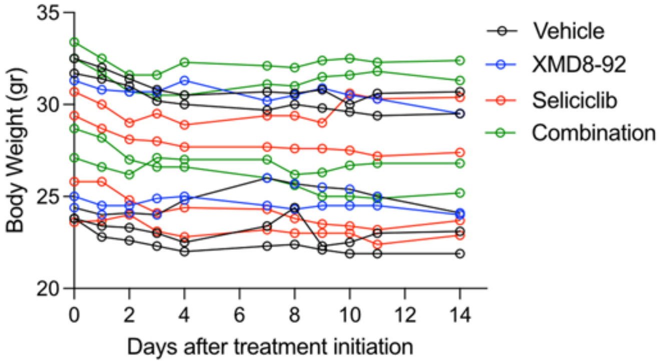

Figure EV3. The treatment of *LSL-Kras$^{G12D/WT}$;p53$^{flox/flox}$* mice with XMD8-92 and/or Seliciclib is well tolerated.

Body weight of *Kras$^{G12D/WT}$;p53$^{flox/flox}$* mice treated with vehicle or XMD8-92 or Seliciclib or combination of XMD8-92 and Seliciclib for 2 weeks.

 © The Author(s)

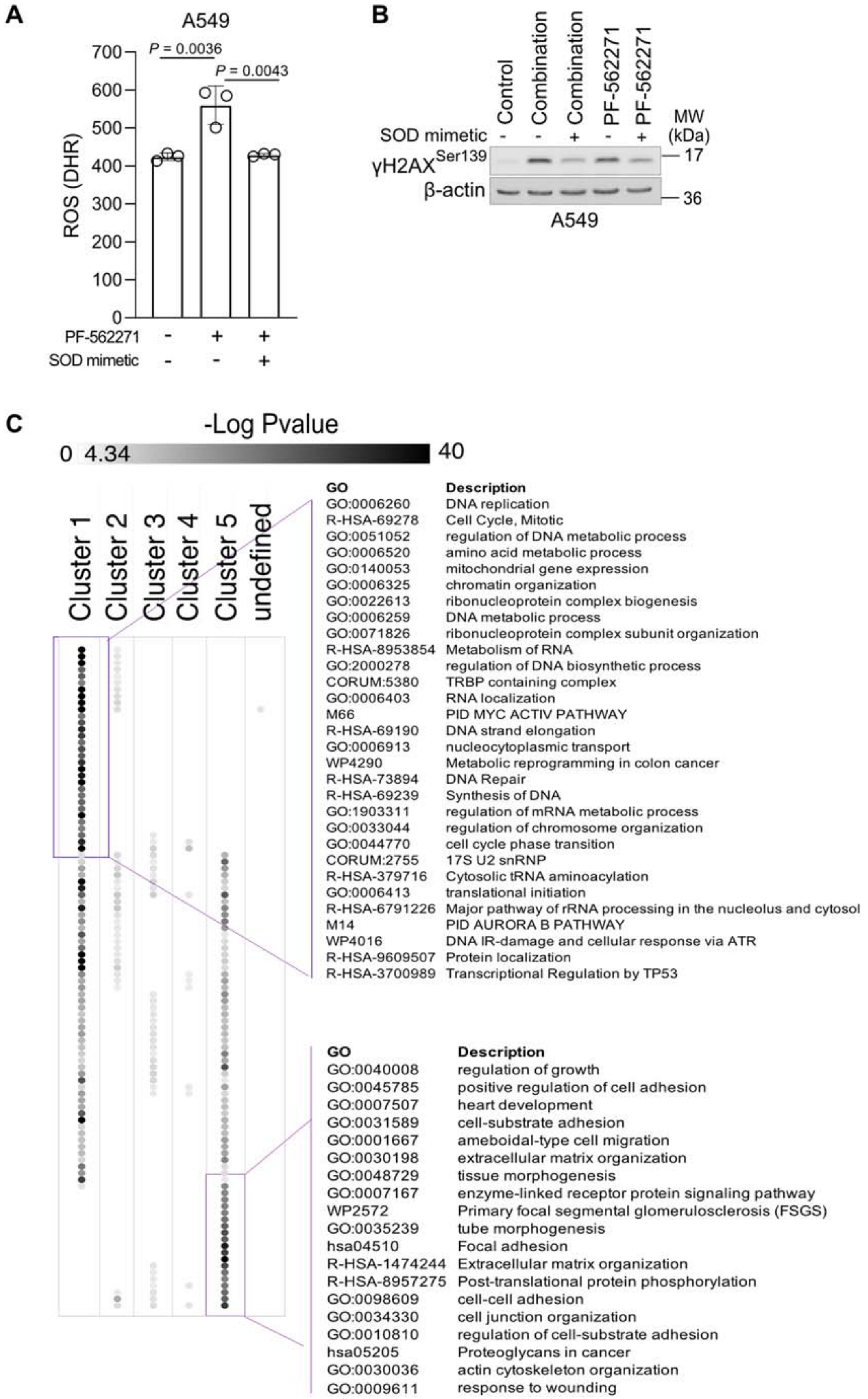
A
A549
P = 0.0036
P = 0.0043
ROS (DHR)
700
600
500
400
300
200
100
0
PF-562271 - + +
SOD mimetic - - +
B
Control
Combination
Combination
PF-562271
PF-562271
SOD mimetic - - + - +
MW (kDa)
γH2AX Ser139
β-actin
17
36
A549
C
-Log Pvalue
0 4.34 40
Cluster 1
Cluster 2
Cluster 3
Cluster 4
Cluster 5
undefined
GO Description
GO:0006260 DNA replication
R-HSA-69278 Cell Cycle, Mitotic
GO:0051052 regulation of DNA metabolic process
GO:0006520 amino acid metabolic process
GO:0140053 mitochondrial gene expression
GO:0006325 chromatin organization
GO:0022613 ribonucleoprotein complex biogenesis
GO:0006259 DNA metabolic process
GO:0071826 ribonucleoprotein complex subunit organization
R-HSA-8953854 Metabolism of RNA
GO:2000278 regulation of DNA biosynthetic process
CORUM:5380 TRBP containing complex
GO:0006403 RNA localization
M66 PID MYC ACTIV PATHWAY
R-HSA-69190 DNA strand elongation
GO:0006913 nucleocytoplasmic transport
WP4290 Metabolic reprogramming in colon cancer
R-HSA-73894 DNA Repair
R-HSA-69239 Synthesis of DNA
GO:1903311 regulation of mRNA metabolic process
GO:0033044 regulation of chromosome organization
GO:0044770 cell cycle phase transition
CORUM:2755 17S U2 snRNP
R-HSA-379716 Cytosolic tRNA aminoacylation
GO:0006413 translational initiation
R-HSA-6791226 Major pathway of rRNA processing in the nucleolus and cytosol
M14 PID AURORA B PATHWAY
WP4016 DNA IR-damage and cellular response via ATR
R-HSA-9609507 Protein localization
R-HSA-3700989 Transcriptional Regulation by TP53
GO Description
GO:0040008 regulation of growth
GO:0045785 positive regulation of cell adhesion
GO:0007507 heart development
GO:0031589 cell-substrate adhesion
GO:0001667 ameboidal-type cell migration
GO:0030198 extracellular matrix organization
GO:0048729 tissue morphogenesis
GO:0007167 enzyme-linked receptor protein signaling pathway
WP2572 Primary focal segmental glomerulosclerosis (FSGS)
GO:0035239 tube morphogenesis
hsa04510 Focal adhesion
R-HSA-1474244 Extracellular matrix organization
R-HSA-8957275 Post-translational protein phosphorylation
GO:0098609 cell-cell adhesion
GO:0034330 cell junction organization
GO:0010810 regulation of cell-substrate adhesion
hsa05205 Proteoglycans in cancer
GO:0030036 actin cytoskeleton organization
GO:0009611 response to wounding

© The Author(s)

Figure EV4. SOD mimetics rescue ROS-induced DNA damage in mutant KRAS NSCLC.

(**A**) Quantification of DHR (ROS marker, green) in A549 cells treated with PF-562271 (10 μM) in the presence or absence of the SOD mimetic, MnTMPyp (25 μM); $n = 3$. Graphical data are mean ± SD. Statistical analyses were done using one-way ANOVA; *n*, number of biologically independent samples. (**B**) Immunoblot analysis for the indicated targets in A549 cells line, treated with DMSO (control) or with a combination of XMD8-92 and Seliciclib (10 μM) or PF-562271 (5 μM) in the presence or absence of the SOD mimetic, MnTMPyp (25 μM). (**C**) Metascape-derived analysis of the functional categories associated to the 5 clusters from main Fig. 5C. Enriched terms were filtered based on the enrichment score and accumulative hypergeometric *P* values ($P < 0.05$). Remaining significant terms were then hierarchically clustered into a tree based on Kappa-statistical similarities among their gene memberships. Then 0.3 kappa score was applied as the threshold to cast the tree into term clusters.

 © The Author(s)

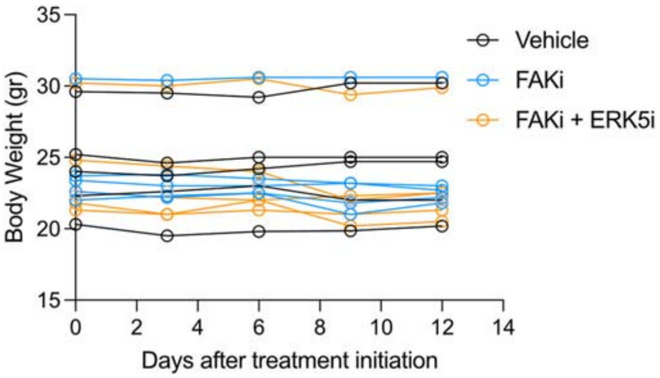

Figure EV5. The treatment of *LSL-Kras*$^{G12D/WT}$*;p53*$^{flox/flox}$ mice with VS4718 (FAKi) or VS4718 (FAKi) + XMD8-92 (ERK5i) is well tolerated.

Body weight of *Kras*$^{G12D/WT}$*;p53*$^{flox/flox}$ mice treated with vehicle or VS-4718 (FAKi) or a combination of VS-4718 and XMD8-92 (FAKi + ERK5i) for 2 weeks.
